# Assessing the Influence of Illumination on Ion Conductivity in Perovskite Solar Cells

**DOI:** 10.1021/acs.jpclett.4c02403

**Published:** 2024-11-03

**Authors:** Andreas Schiller, Sandra Jenatsch, Balthasar Blülle, Miguel Angel Torre Cachafeiro, Firouzeh Ebadi, Nasim Kabir, Mostafa Othman, Christian Michael Wolff, Aïcha Hessler-Wyser, Christophe Ballif, Wolfgang Tress, Beat Ruhstaller

**Affiliations:** † Fluxim AG, Katharina-Sulzer-Platz 2, 8400 Winterthur, Switzerland; ‡ Institute of Computational Physics, Zurich University of Applied Sciences (ZHAW), Technikumstrasse 71, 8401 Winterthur, Switzerland; ¶ Photovoltaics and Thin-Film Electronics Laboratory (PV-Lab), Institute of Electrical and Micro Engineering (IEM), École Polytechnique Fédérale de Lausanne (EPFL), Rue de la Maladière 71b, 2002 Neuchâtel, Switzerland

## Abstract

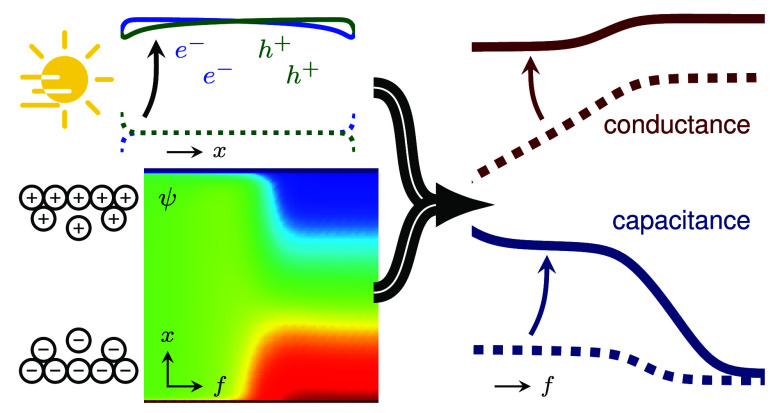

Whether illumination influences the ion conductivity in lead-halide perovskite solar cells containing iodide halides has been an ongoing debate. Experiments to elucidate the presence of a photoconductive effect require special devices or measurement techniques and neglect possible influences of the enhanced electronic charge concentrations. Here, we assess the electronic-ionic charge transport using drift-diffusion simulations and show that the well-known increase in capacitance at low frequencies under illumination is caused by electronic currents that are amplified due to the screening of the alternating electric field by the ions. We propose a novel characterization technique to detect a potential photoinduced increase in ionic conductivity based on capacitance measurements on fully integrated devices. The method is applied to a range of perovskite solar cells with different active layer materials. Remarkably, all measured samples show a clear signature of photoenhanced ion conductivity, posing fundamental questions on the underlying nature of the photosensitive mechanism.

Electrochemical impedance spectroscopy (EIS) studies of perovskite solar cells (PSCs) have reported a large impact of the illumination intensity on the capacitance at low frequencies.^1,2^ This effect was first ascribed to an increase in the dielectric constant,^1^ later to an illumination-induced accumulation of ions at the interfaces^2,3^ or to a light-induced reduction of the activation energy of ions.^4,5^ The latter was supported by various experiments but interpreted under the assumption of independent electronic and ionic charge transport. An increase in defects and thus the density of states of the ions would cause a similar influence on the ion conductivity. This might be caused directly by the illumination^6−8^ or induced by accumulating free charge carriers.^9−11^

Challenging these hypotheses, Jacobs et al.^12^ showed that the measured variation of the capacitance can be reproduced using drift-diffusion simulations. They attributed the effects to ionically modulated injection and recombination and thus argued, that they emerge naturally as a consequence of ion migration. This was supported by a model ascribing the low-frequency effects on impedance to modulated electronic interface recombination^13^ and a study linking the time-scales of these effects on the capacitance to the ion-induced hysteresis in transient current–voltage measurements.^14^

Drift-diffusion models have proven to be viable for the understanding of mixed electronic-ionic transport in general^15^ and PSCs in particular.^16−18^ Also, drift-diffusion simulations of EIS that complement the established models by mixed electronic-ionic interaction are well established to qualitatively and quantitatively reproduce measurement results such as multiple arcs in the Cole–Cole diagram of the impedance, an increase in capacitance at low frequencies, and negative capacitance.^13,18−24^

In this letter, we will first investigate the origin of the illumination-dependent low-frequency behavior in EIS. This investigation starts with triple cation PSCs using the stack FTO/c-TiO2/mp-TiO2/Cs_0.05_(MA_0.17_FA_0.83_)_0.95_ Pb(I_0.83_Br_0.17_)_3_/Spiro/Au.^25^ The results of EIS measurements at short-circuit of such a PSC down to mHz frequencies in the dark and under 0.1 sun illumination are shown in Figures 1(a) and (c) and show the known characteristic features.

**Figure 1:**
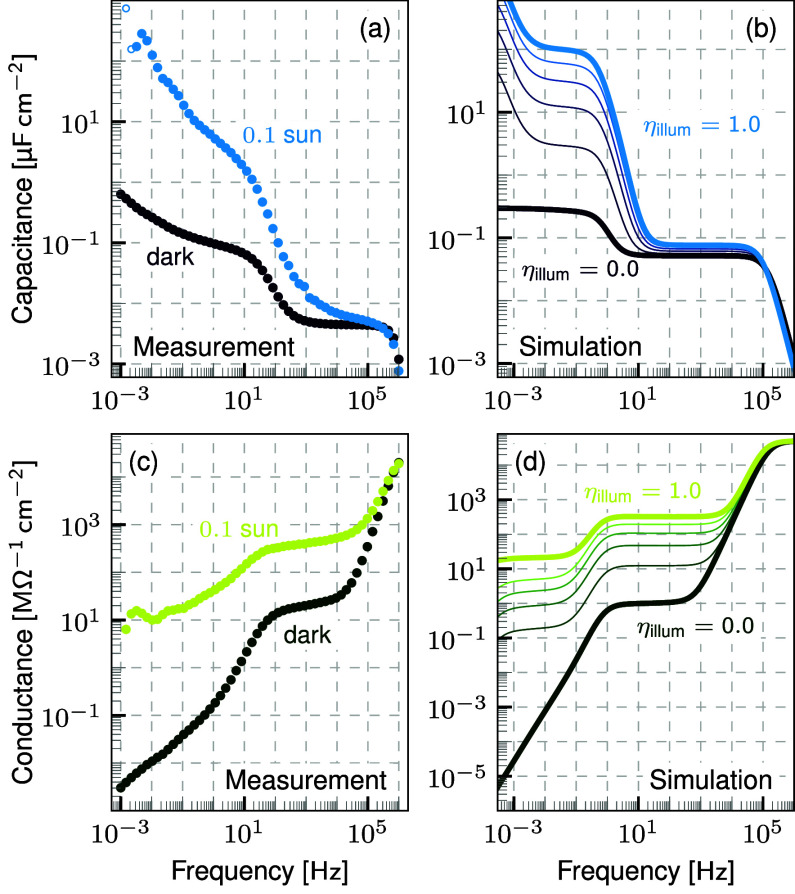
Illumination dependent capacitance and conductance at short-circuit (a),(c) measured on a triple cation PSC and (b),(d) simulated by Setfos using a simple device model of a mixed electronic-ionic conductor active material sandwiched between two doped transport layers. The measured data is qualitatively well reproduced by the simulation results. The capacitance shows an increase toward low frequencies which drastically increases under illumination while the onset frequency increases as well. The conductance shows an additional plateau in the Hz to kHz range in the dark and a decrease toward low frequencies with increasing magnitude under illumination.

The measured results are reproduced by drift-diffusion simulations performed with Fluxim’s simulation software Setfos 5.5 as shown in Figures 1(b) and (d). Keeping the device structure simple and generic, the active layer of perovskite is modeled as a mixed electronic-ionic conductor surrounded by two doped transport layers. Disparate constant mobilities are assumed for the two ion species in the active layer. The optical generation is modeled by a constant generation profile in the perovskite layer for simplicity. The drift-diffusion model equations and device parameters are provided in the Supporting Information. Since the device is symmetric, the ion species are interchangeable. Any more advanced models are intentionally excluded to focus solely on the effects of the interaction between ionic and electronic charge carriers in the presence of an illumination intensity variation.

As mentioned in the first two paragraphs, the origin of the characteristic low-frequency response has been the topic of debate. The frequency range of the capacity increase suggests a contribution by the slow ionic charge carriers,^14^ while its sensitivity to illumination hints toward an electronic mechanism, as in the model only the electronic charge carriers are directly linked to the illumination intensity via the optical generation. Additional effects such as an illumination-dependent dielectric constant, activation energy, or increase in defects can be ruled out as cause for the illumination-dependent capacitance in the simulation, since they are not included in the present model used to reproduce the measurement results.

In a first step, the origin of the alternating current responsible for the effects is investigated. To do so, the parallel capacitance and conductance can be expressed as the sum of the contributions by the different charge carrier currents and the displacement current:
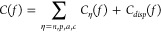
1a
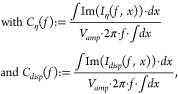

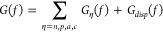
1b
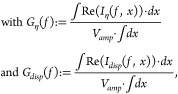
where *I*_η_(*f*, *x*) are the alternating charge carrier currents and *I*_*disp*_(*f*, *x*) is the alternating displacement current at frequency *f* and position *x*. *V*_*amp*_ is the amplitude of the applied alternating voltage. The derivation of these equations is outlined in the Supporting Information. In simulation, these contributions can be computed and provide insight into the origin of the overall results. Figure 2 shows the contributions for the drift-diffusion simulation results. As expected, the capacitance increase at low frequencies and the plateau in conductance in the dark are caused by ionic charge carrier currents. However, these contributions remain unaltered under illumination and the illumination-dependency is instead caused by the contributions of the electron and hole currents for both the capacitance and conductance.

**Figure 2:**
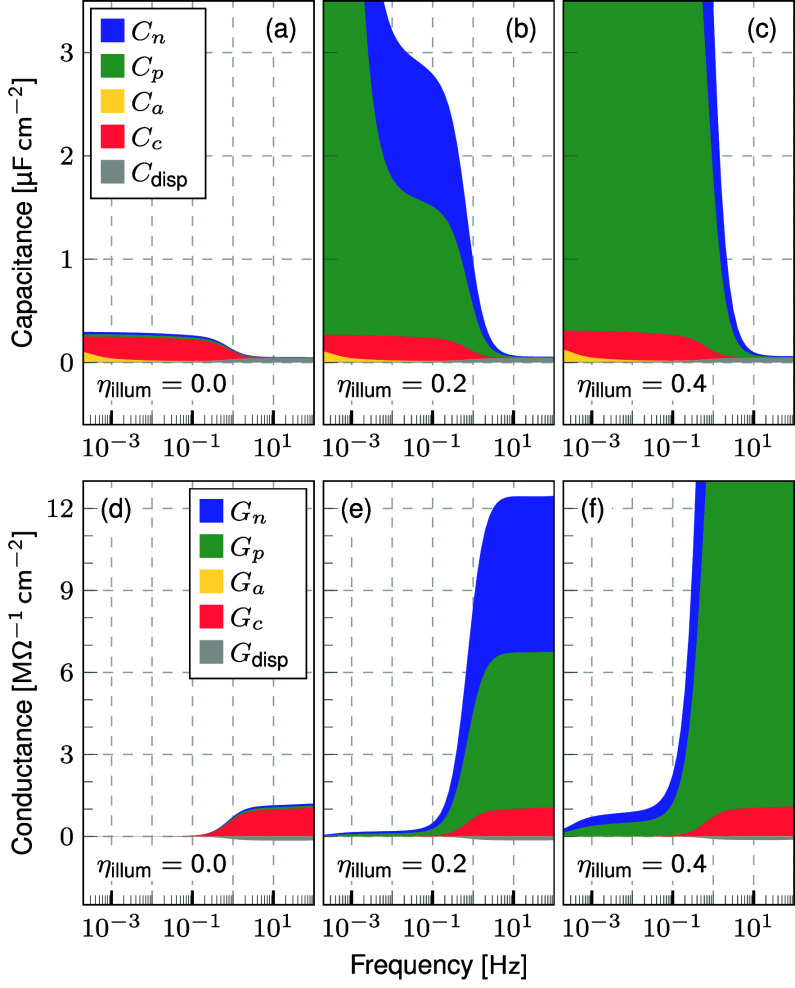
Contribution by charge carrier and displacement currents to (a)-(c) capacitance and (d)-(f) conductance as introduced in eqs 1a and 1b at varying illumination intensity. The contributions by the ions are independent from the illumination while the dominant, characteristic features are caused by contributions from the electron and hole currents. It is worth mentioning that the contributions by the electron and hole current are equal due to the symmetry of the modeled device structure.

It might be surprising, that the contributions by the electronic charge carrier currents show a distinct feature around 1 Hz, as this does not correspond to the much higher characteristic frequency of electrons and holes. On the contrary, the dynamics of the electronic contribution matches the characteristic frequency of the cations, as can be seen by the cation current contribution showing a feature at the same frequency. To explain this correlation, it has to be considered that the superpositions in eqs 1a and 1b do not reflect the full nonlinear dynamics occurring in the semiconducting layers of the device. Due to the coupling among the charge carriers mediated by the electrostatic potential, modifying a parameter of one charge carrier will usually affect all currents and thus all contributions to capacitance and conductance.

To investigate these interdependencies, we take a closer look at the influence of the ions on the overall results. To do so, one could ignore the ionic contribution in the simulation and compare the results with and without considering mobile ions. However, applying this approach to EIS simulations, the influences of the excluded ionic charges on the AC simulation and on the steady-state might not be easily distinguishable, because the small-signal AC response is always based on the specific steady-state and thus dependent on the latter. An elegant approach suggested here is to include the mobile ions in the steady-state simulation but treat their distribution as a static background charge, like e.g. a doping density, within the AC simulation. Doing this for both ion species results in the capacitance and conductance shown in Figure 3(a) and (b). They reveal that the illumination-dependent effects depend on the oscillating ionic charge carriers and vanish if the latter are kept static. Together with the analysis of the current contributions, this leads to the conclusion that the light intensity modulation of the low-frequency capacitance and conductance is caused by the oscillating ionic charge carriers influencing the alternating electron and hole currents.

**Figure 3:**
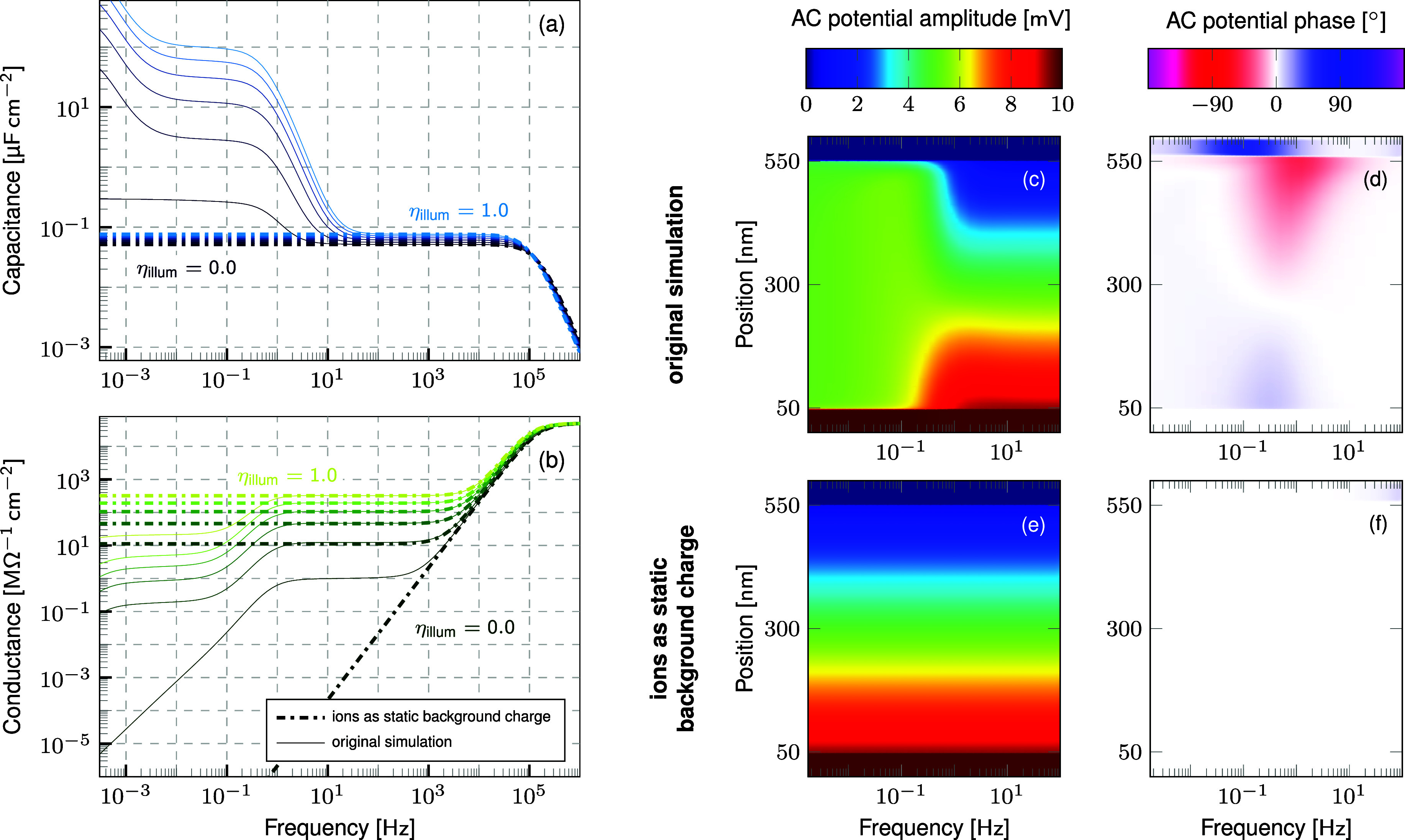
(a) Capacitance and (b) conductance obtained by an AC simulation treating the ions as static background charge (dashed lines) based on the same steady-state as the original simulation (solid lines), which is shown for comparison. The difference between the two simulations originates from the oscillating ionic charge carriers. (c),(e) Amplitude and (d),(f) phase of the alternating potential at illumination intensity prefactor η_*illu*_ = 1.0 of (c)-(d) the original simulation and (e)-(f) the simulation treating the ions as static background charge. The screening of the alternating electric field below 1 Hz visible in (c) and the phase shift visible in (d) are caused by the oscillating ions and are thus missing in (e) and (f), respectively.

As the only interaction between electronic and ionic charges is through the alternating electrostatic potential, we can compare the potential profiles for the situations with static and dynamic ions, respectively. The results shown in Figure 3(c)−(f) reveal how the oscillating ionic charge carriers screen the alternating electric field as soon as the frequency is low enough to allow the ions to accumulate at the interfaces and disperse again within one oscillation. This suppression of the alternating electric field reduces the alternating electronic charge carrier drift currents and consequently the conductance. The increase in capacitance is caused by the phase shift initiated by the ionic charge carriers and inherited by the electronic charge carriers since their oscillations are slowed due to the modulated screening of the alternating electric field. In contrast to previous studies,^12,13^ the recombination is not causative for the large capacitance at low frequencies for the studied devices, as the effects do not vanish even if no recombination mechanisms are considered (Figure S7(e) in the Supporting Information). However, recombination significantly increases the magnitude of the effects.

With this, the investigation on the origin of the large, photoinduced effects at low frequencies can be concluded. It has been shown, that the measurement results can be qualitatively reproduced by a simple drift-diffusion model of a mixed electronic-ionic conductor. While the origin of the enhanced capacitance in the dark can be traced back to ionic charge carrier currents, the illumination-induced enhancement is caused by electronic charge carrier currents which are altered by the screening of the alternating electric field by the oscillating ions. Still, this does not disprove a possible photoconductive effect of the ions. Especially, as this has been reported multiple times and was supported experimentally using different methods beyond EIS.^4,5,7,8^ Thus, in the following, the drift-diffusion model will be used to investigate how a photoconductive effect would impact the EIS results.

Lacking a comprehensive model for a possible photoconductivity of ions, the ion conductivity has to be artificially scaled with the illumination intensity. In the drift-diffusion model, this can be achieved by scaling the ion mobility or concentration. For this analysis, the ion mobilities or concentrations are increased by one order of magnitude while the illumination intensity prefactor is increased from 0 to 1. The applied formula is given in the Supporting Information in eqs S6a and b. This modification results in slightly different EIS characteristics as shown in Figure 4(a)-(c), but the overall qualitative behavior is conserved. Assessing the presence or absence of a photoconductive effect based on these curves alone would require a quantitatively fitted model for every new device structure. However, closer examination reveals a qualitative difference in the frequency shift of the onset in capacitance at short-circuit. This is the basis of the characterization technique discussed hereinafter.

**Figure 4:**
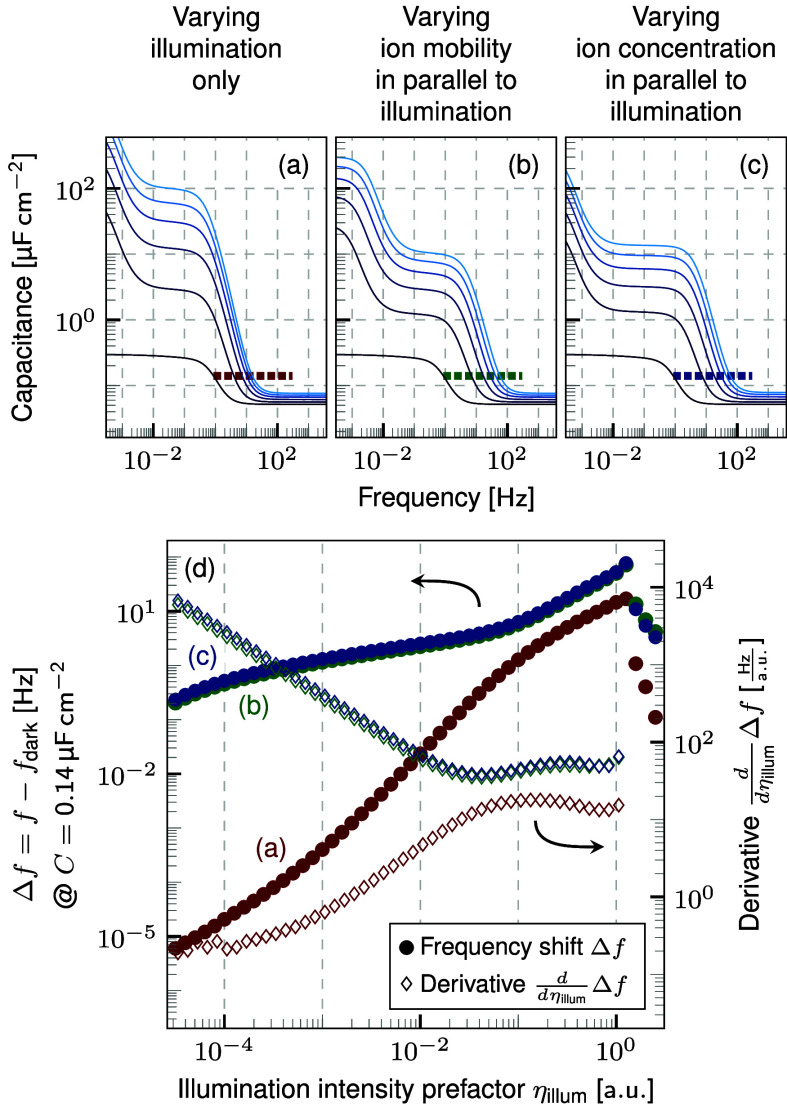
Capacitance obtained for (a) the original simulation and the same simulation but additionally increasing the (b) ion mobility or (c) ion concentration, respectively, in parallel to the illumination. Despite the quantitative difference, an assessment of the presence of a photoconductive effect is not possible in this data representation. (d) Frequency shift of the capacitance onset and its derivative for the three cases (a) in red, (b) in green, and (c) in blue. The green markers are almost completely covered by the blue ones, as increasing the ion mobility and concentration have a very similar impact on the results. Both the frequency shift and its derivative show a distinct qualitative difference depending on the presence of a photoconductive effect.

For this analysis, the frequency at which the capacitance reaches a certain value is extracted. The choice of the capacitance value between the two plateaus at lower and higher frequencies does not affect the qualitative results as shown in Figure S4 in the Supporting Information.

Plotting the difference in the extracted frequency at different illumination intensities and in the dark reveals a distinct qualitative difference as shown in Figure 4(d). Starting from the point of reference at η_*illum*_ ≈ 1.0, all three curves decrease with a constant derivative for approximately one and a half orders of magnitude. At lower illumination intensities, the derivative obtained from the simulation without a photoconductive effect on the ions decreases. In clear contrast, the derivatives of the other two simulations increase with decreasing illumination intensity.

Applying this method requires a series of capacitance measurements over several orders of magnitude in illumination intensity to capture both, the decrease with a constant derivative and the decreasing or increasing derivative at lower illumination intensities. Therefore, choosing the range of illumination intensity is relevant, as both the absolute value and the slope of the frequency shift depend on the device structure and material parameters. Ideally, the illumination intensity at which the capacitance becomes negative and the extracted frequencies become smaller with increasing illumination intensity can be used as the point of reference (see Figure S5 in the Supporting Information). But as will be shown later, the method can also work without a point of reference.

We find that the onset of the capacitance increase is a favorable characteristic feature, as it occurs at a relatively large frequency, whereas in the lower frequency regime, the measurements become slower and noisier. EIS measurements on four PSCs with different active materials were performed using the Paios measurement platform that includes a white LED light source which is linearly calibrated. The measured frequency shift and its derivative are shown in Figure 5. The underlying capacitance measurements are provided in Figure S6 in the Supporting Information.

**Figure 5:**
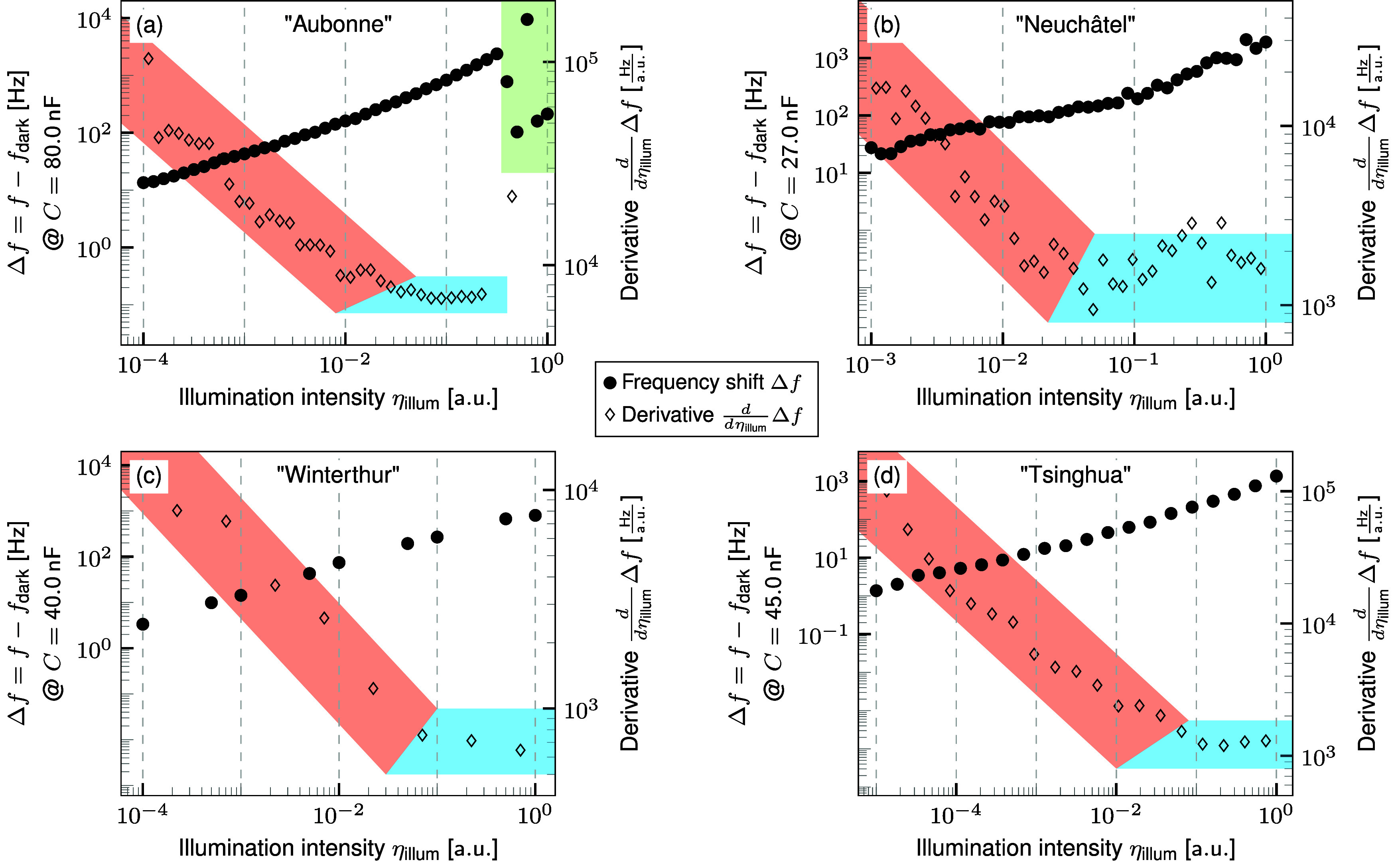
Frequency shift of the capacitance onset and its derivative for (a) a PSC with a carbon-based back-electrode, (b) a double cation PSC, (c) a triple cation PSC, and (d) a FAPbI PSC. The green area highlights the illumination intensities at which the capacitance becomes negative. The blue area marks the range in which the derivative remains constant with illumination intensity before it increases toward lower illumination intensities within the red area. The measurement results from all devices qualitatively agree with the results obtained by simulations that mimic a photoconductive effect (Figure 4).

The device structure of the PSC ”Aubonne” with a carbon-based back-electrode is described by Bogachuk et al.^26^ Its measurement results are shown in Figure 5(a). The point of reference for this cell lies at an illumination intensity of approximately 0.35. As expected, the derivative then remains constant for approximately one and a half orders of magnitude. The derivative increases below an illumination intensity of 10^–2^, which coincides with the simulation results that mimic a photoconductive effect.

Depending on the properties of the solar cell, the capacitance might not become negative at high illumination intensities. This seems to be the case in the example of the double cation PSC ”Neuchâtel” shown in Figure 5(b). Its device stack is comprised of indium–tin oxide (ITO)-coated glass/(4-(3,6-dimethyl-9H-carbazol-9-yl)butyl)phosphonic acid (Me-4PACz)/silicon-oxide nanoparticles (SiOx-NPs)/Cs0.17FA0.83PbI3/buckminsterfullerene (C60)/Tin(IV) Oxide (SnOx)/copper (Cu). Instead of the decrease due to the negative capacitance values, the derivative of the frequency shift increases toward higher illumination intensities for this cell. However, the derivative still shows a plateau over approximately one and a half orders of magnitude in illumination intensity and an increase toward lower illumination intensities.

Limitations by the measurement equipment or degradation of the solar cell under high illumination intensities might inhibit measuring up to the point of reference. The measurement results of the triple cation PSC ”Winterthur” are shown in Figure 5(c). It consists of FTO/c-TiO2/ mp-TiO2/Cs_0.05_(MA_0.17_FA_0.83_)_0.95_Pb(I_0.83_Br_0.17_)_3_/Spiro/Au. The measurement results of the FAPbI PSC ”Tsinghua” are shown in Figure 5(d). It is formed of FTO/SnO2/FAPbI3/Spiro/Au. Despite the missing point of reference and a low resolution of the illumination intensity, both show the increase of the derivative toward lower illumination intensities expected for materials with a photoconductive effect of the ions.

Remarkably, all four measured PSCs show the frequency shift characteristics expected for a material with an illumination-dependent ion conductivity.

After demonstrating a simple drift-diffusion model of a mixed electronic-ionic conductor that reproduces the photoinduced effects observed in EIS measurements of PSCs, a novel measurement protocol to assess a possible photoconductive effect on the ions has been introduced. Based on the qualitative shape of the illumination-dependent frequency shift in capacitance measurements of fully integrated devices, the presence of a photoconductive effect on the ions can be detected. The method was applied to four different PSCs containing iodide halides and confirmed the presence of a photoconductive effect in all devices. These findings are in line with previous reports,^4,5,7,8^ but pose a challenge from a modeling perspective. Directly linking the ion mobility or concentration to the external illumination intensity reproduces the measurement results, but lacks a physical model to explain this correlation. Besides the obvious problem this poses for illumination-dependent simulations, the missing model extension might also contain missing interrelations between the ion conductivity and other physical parameters.

Thus, in future work, the artificial increase in ion conductivity needs to be replaced by a comprehensive model. At present, we can only speculate about the origin of this missing link between illumination intensity and ion conductivity, but since a direct interaction of light with ions lacks a physical foundation, the coupling is most likely mediated by the increased electron and hole density caused by the higher number of absorbed photons. Singh et al.^27^ described a model extension in which mobile ions act as trap states. However, since in their model, the charged ions become neutral by trapping an electron or hole, the ion concentration would decrease with an increasing electron and hole concentration. Based on the chemical processes discussed by Bitton and Tessler,^28^ a possible pathway is a trapping process in which initially neutral and mobile species trap electrons or holes to become mobile ions. This model would explain the increasing concentration of mobile ions with increasing illumination intensity.

## Supporting Information Available

Drift-diffusion model equations and device parameters, Derivation of capacitance and conductance components, Illumination-dependent steady-state profiles, Alternating potential profiles in the dark, Choice of capacitance at which to extract the frequency, Negative capacitance at high illumination intensity, Capacitance and conductance measurement results, and Influence of recombination (PDF)
Transparent Peer Review report available (PDF)
