# Supplementary material for: Assessing the Influence of Illumination on Ion Conductivity in Perovskite Solar Cells
Source: J Phys Chem Lett. 2024 Nov 3;15(45):11252–8. doi: 10.1021/acs.jpclett.4c02403 (PMC11571209; doi:10.1021/acs.jpclett.4c02403)
Supplement: Supplementary file 1 — jz4c02403_si_001.pdf [file jz4c02403_si_001.pdf]

Supporting Information: Assessing the Influence of Illumination on Ion Conductivity in Perovskite Solar Cells

Andreas Schiller,[*,†,‡] Sandra Jenatsch,[†] Balthasar Blülle,[†]
Miguel Angel Torre Cachafeiro,[‡] Firouzeh Ebadi,[‡] Nasim Kabir,[‡]
Mostafa Othman,[¶] Christian Michael Wolff,[¶] Aïcha Hessler-Wyser,[¶]
Christophe Ballif,[¶] Wolfgang Tress,[‡] and Beat Ruhstaller[†,‡]

†*Fluxim AG, Katharina-Sulzer-Platz 2, 8400 Winterthur, Switzerland*

‡*Institute of Computational Physics, Zurich University of Applied Sciences (ZHAW), Technikumstrasse 71, 8401 Winterthur, Switzerland*

¶*Photovoltaics and Thin-Film Electronics Laboratory (PV-Lab), Institute of electrical and micro engineering (IEM), École polytechnique fédérale de Lausanne (EPFL), Rue de la Maladière 71b, 2002 Neuchâtel, Switzerland*

E-mail: andreas.schiller@fluxim.com

Drift-diffusion model equations and device parameters

All drift-diffusion simulations were performed with Fluxim's simulation software Setfos 5.5, which solves the one-dimensional drift-diffusion equations in steady-state, transient, and frequency-domain.[S1]

The drift-diffusion equations consist of Poisson's equation for the electrostatic potential

$$\nabla \cdot (\varepsilon(x) \cdot \nabla \psi(x)) = -\rho(x)\,, \tag{S1}$$

where $\varepsilon(x)$ is the electrical permittivity of the material, $\psi(x)$ is the electrostatic potential, and $\rho(x)$ is the net charge density and the current continuity equations[S2]

$$\nabla \cdot J_e(x) - q \cdot \frac{\partial n_e(x)}{\partial t} = q \cdot (R(x) - \eta_{\text{illum}} \cdot G(x)) \tag{S2a}$$
$$\text{with } J_e(x) = -n_e(x) \cdot \mu_e(x) \cdot \nabla \psi(x) + kT\mu_e(x) \cdot \nabla n_e(x)\,,$$
$$\nabla \cdot J_h(x) + q \cdot \frac{\partial n_h(x)}{\partial t} = -q \cdot (R(x) - \eta_{\text{illum}} \cdot G(x)) \tag{S2b}$$
$$\text{with } J_h(x) = n_h(x) \cdot \mu_h(x) \cdot \nabla \psi(x) + kT\mu_h(x) \cdot \nabla n_h(x)\,,$$
$$\nabla \cdot J_a(x) - q \cdot \frac{\partial n_a(x)}{\partial t} = 0 \tag{S2c}$$
$$\text{with } J_a(x) = -n_a(x) \cdot \mu_a(x) \cdot \nabla \psi(x) + kT\mu_a(x) \cdot \nabla n_a(x)\,,$$
$$\nabla \cdot J_c(x) + q \cdot \frac{\partial n_c(x)}{\partial t} = 0 \tag{S2d}$$
$$\text{with } J_c(x) = n_c(x) \cdot \mu_c(x) \cdot \nabla \psi(x) + kT\mu_c(x) \cdot \nabla n_c(x)\,,$$

where $J(x)$ is the charge carrier current, q is the elementary charge, $n(x)$ is the charge carrier density, $R(x)$ is the recombination, η_{illum} is the illumination intensity prefactor, $G(x)$ is the generation, $\mu(x)$ is the charge carrier mobility, $\psi(x)$ is the electrostatic potential, k is the Boltzmann constant, T is the temperature, and t is the time. The subscripts denote the charge carrier types n for electrons, h for holes, a for anions, and c for cations.

The recombination is modeled as Langevin recombination,[S3] which is often used to model

organic semiconductors[S4] and keeps the number of parameters to a minimum.

$$R(x) = \eta_{\mathrm{Lang}}(x) \cdot \frac{q}{\varepsilon(x)} \cdot (\mu_e(x) + \mu_h(x)) \cdot \left(n_e(x) \cdot n_h(x) - n_{\mathrm{intr}}^2\right) \ , \tag{S3}$$

where η_{Lang} is the Langevin recombination efficiency and n_{intr} is the geometric average of the carrier concentrations in equilibrium. Due to the constant mobility, this is equivalent to a bimolecular recombination model with the respective prefactor.

The boundary conditions for the electron and hole densities are assumed to follow Ohm's law at the electrodes and the quasi-Fermi level continuity at the interfaces.[S5]

$$n_e(x_0) = N_e(x_0) \cdot \exp\left(\frac{E_{\mathrm{LUMO}} - W}{kT}\right) \ , \tag{S4a}$$

$$n_h(x_0) = N_h(x_0) \cdot \exp\left(\frac{W - E_{\mathrm{HOMO}}}{kT}\right) \ , \tag{S4b}$$

$$E_{\mathrm{LUMO}}(x_\ell) - kT \cdot \ln\left(\frac{N_e(x_\ell)}{n_e(x_\ell)}\right) = E_{\mathrm{LUMO}}(x_r) - kT \cdot \ln\left(\frac{N_e(x_r)}{n_e(x_r)}\right) \ , \tag{S4c}$$

$$E_{\mathrm{HOMO}}(x_\ell) + kT \cdot \ln\left(\frac{N_h(x_\ell)}{n_h(x_\ell)}\right) = E_{\mathrm{HOMO}}(x_r) + kT \cdot \ln\left(\frac{N_h(x_r)}{n_h(x_r)}\right) \ , \tag{S4d}$$

where x_0 is the position of the electrode, $N_e(x)$ and $N_h(x)$ are the density of states for the electrons and holes respectively, E_{LUMO} is the energy of the conduction band, E_{HOMO} is the energy of the valence band, W is the workfunction of the electrode, x_ℓ is the position of the interface within the left-hand layer, and x_r is the position of the interface within the right-hand layer.

The ions are assumed to be confined within the perovskite layer. Lacking any gain or loss mechanisms, the total number of charge carriers is conserved.

$$J_a(x_r) = J_a(x_\ell) = J_c(x_r) = J_c(x_\ell) = 0 \, , \tag{S5a}$$

$$\int_{x_r}^{x_\ell} n_a(x) \cdot dx = \int_{x_r}^{x_\ell} n_{\mathrm{initial},a}(x) \cdot dx \, , \tag{S5b}$$

$$\int_{x_r}^{x_\ell} n_c(x) \cdot dx = \int_{x_r}^{x_\ell} n_{\mathrm{initial},c}(x) \cdot dx \, , \tag{S5c}$$

where $n_{\text{initial},a}$ and $n_{\text{initial},c}$ are the initial anion and cation densities given as material parameters. Equations S5b and S5c are only needed in the steady-state system of equations, as in transient and frequency-domain equation S5a is sufficient to conserve the overall ion density.

The generic device used throughout the publication evolved from the fitted parameter set obtained by Neukom et al.[S6] and was simplified for the purpose of this work. Its device structure, energy diagram, and material parameters are given in figure S1.

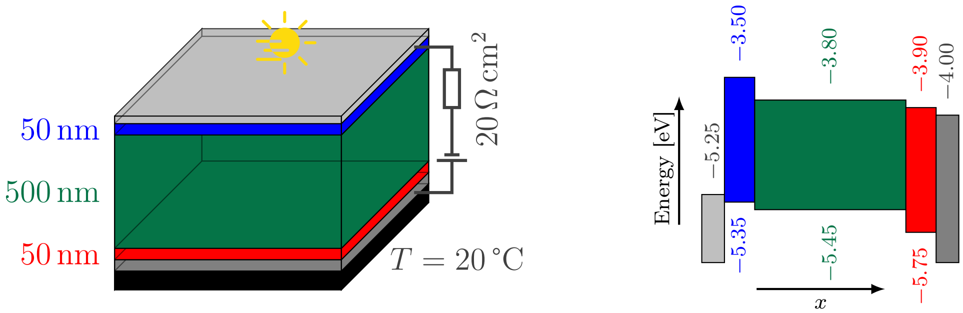

Parameter	HTL	Perovskite	ETL
rel. elec. permittivity ε_r	3	35	3
density of states $N_{0,e}$	$1 \cdot 10^{21}\,\text{cm}^{-3}$	$5 \cdot 10^{19}\,\text{cm}^{-3}$	$1 \cdot 10^{21}\,\text{cm}^{-3}$
density of states $N_{0,h}$	$1 \cdot 10^{21}\,\text{cm}^{-3}$	$5 \cdot 10^{19}\,\text{cm}^{-3}$	$1 \cdot 10^{21}\,\text{cm}^{-3}$
electron mobility μ_e	$1 \cdot 10^{-3}\,\text{cm}^2\,\text{V}^{-1}\,\text{s}^{-1}$	$1\,\text{cm}^2\,\text{V}^{-1}\,\text{s}^{-1}$	$1 \cdot 10^{-3}\,\text{cm}^2\,\text{V}^{-1}\,\text{s}^{-1}$
hole mobility μ_h	$1 \cdot 10^{-3}\,\text{cm}^2\,\text{V}^{-1}\,\text{s}^{-1}$	$1\,\text{cm}^2\,\text{V}^{-1}\,\text{s}^{-1}$	$1 \cdot 10^{-3}\,\text{cm}^2\,\text{V}^{-1}\,\text{s}^{-1}$
acceptor doping N_a	$1 \cdot 10^{19}\,\text{cm}^{-3}$	–	–
donor doping N_d	–	–	$1 \cdot 10^{19}\,\text{cm}^{-3}$
initial anion density $n_{\text{initial},a}$	–	$5 \cdot 10^{17}\,\text{cm}^{-3}$	–
anion mobility μ_a	–	$1 \cdot 10^{-11}\,\text{cm}^2\,\text{V}^{-1}\,\text{s}^{-1}$	–
initial cation density $n_{\text{initial},c}$	–	$5 \cdot 10^{17}\,\text{cm}^{-3}$	–
cation mobility μ_c	–	$1 \cdot 10^{-9}\,\text{cm}^2\,\text{V}^{-1}\,\text{s}^{-1}$	–
optical generation G	–	$5 \cdot 10^{21}\,\text{cm}^{-3}\,\text{s}^{-1}$	–
Langevin recomb. eff. η_{Lang}	1.0	0.001	1.0
mesh element size in layer bulk	2 nm	4 nm	2 nm
mesh element size within 10 nm of the interfaces	–	0.5 nm	–

Figure S1: Device structure, energy diagram, and parameters for the generic device used for the drift-diffusion simulations.

To model a possible photo-conductive effect, either the ion mobilities or initial ion densities are modified depending on the illumination intensity prefactor:

$$\mu = \mu^{\text{orig}} \cdot (1 + 9 \cdot \eta_{\text{illum}}) \tag{S6a}$$

$$n_{\text{initial}} = n_{\text{initial}}^{\text{orig}} \cdot (1 + 9 \cdot \eta_{\text{illum}}) \tag{S6b}$$

Derivation of capacitance and conductance components

The capacitance and conductance components introduced in equations 1a and 1b computed from the results of a drift-diffusion simulation are derived from the components of the device current

$$I(t) = I(x,t) = \sum_{\eta=e,h,a,c} I_\eta(x,t) + I_{\mathrm{disp}}(x,t)\,, \tag{S7}$$

where $I(t) = I(x,t)$ is the device current which is constant over the position x, $I_\eta(x,t)$ is the charge carrier current, and $I_{\mathrm{disp}}(x,t)$ is the displacement current.

In the small-signal impedance simulation, a sinusoidal voltage signal

$$V(f,t) = V_0 + \mathrm{Re}\left(V_{\mathrm{amp}} \cdot e^{i\omega t}\right)\,, \tag{S8}$$

is applied where V_0 is the applied steady-state voltage, V_{amp} is the real amplitude, and $\omega = 2\pi f$ is the angular frequency.

The resulting currents are assumed to be sinusoidal as well. The phase shift with respect to the applied voltage is accounted for by the phase of the complex amplitude.

$$\begin{aligned}
I(f,x,t) =& I_0 + \sum_{\eta=e,h,a,c} \mathrm{Re}\left(I_\eta(x,f)\cdot e^{i\omega t}\right) + \mathrm{Re}\left(I_{\mathrm{disp}}(x,f)\cdot e^{i\omega t}\right)\\
=& I_0 + \mathrm{Re}\left(\left(\sum_{\eta=e,h,a,c} I_\eta(x,f) + I_{\mathrm{disp}}(x,f)\right)\cdot e^{i\omega t}\right)\\
=& I_0 + \mathrm{Re}\left(I(x,f)\cdot e^{i\omega t}\right)\\
=& I_0 + \mathrm{Re}\left(I(f)\cdot e^{i\omega t}\right)
\end{aligned} \tag{S9}$$

where $I_\eta(x,f)$ and $I_{\mathrm{disp}}(x,f)$ are the complex amplitudes of the position-dependent charge carrier and displacement currents and I_0 is the steady-state current. The resulting device current $I(x) = I(x,f)$ is again constant over position x.

Integrating the current components over the position, the complex current amplitude can

be expressed as the sum of five current components.

$$I(f) = \frac{\int I(x,f)dx}{\int dx} = \sum_{\eta=e,h,a,c} \frac{\int I_\eta(x,f)dx}{\int dx} + \frac{\int I_{\text{disp}}(x,f)dx}{\int dx} \tag{S10}$$

The impedance $Z(f)$ and the admittance $Y(f)$ are then computed from the current and voltage amplitudes.

$$Z(f) = \frac{V_{\text{amp}}}{I(f)} \tag{S11a}$$

$$Y(f) = \frac{1}{Z(f)} = \frac{I(f)}{V_{\text{amp}}} \tag{S11b}$$

The parallel capacitance $C(f)$ and conductance $G(f)$ are defined in terms of the real and imaginary part of the admittance.[S7]

$$C(f) = \frac{1}{\omega} \cdot \text{Im}\left(Y(f)\right) = \frac{1}{\omega} \cdot \text{Im}\left(\frac{I(f)}{V_{\text{amp}}}\right) \tag{S12a}$$

$$G(f) = \text{Re}\left(Y(f)\right) = \text{Re}\left(\frac{I(f)}{V_{\text{amp}}}\right) \tag{S12b}$$

Inserting the expression for $I(f)$ from equation S10 results in equations 1a and 1b in the publication.

Illumination-dependent steady-state profiles

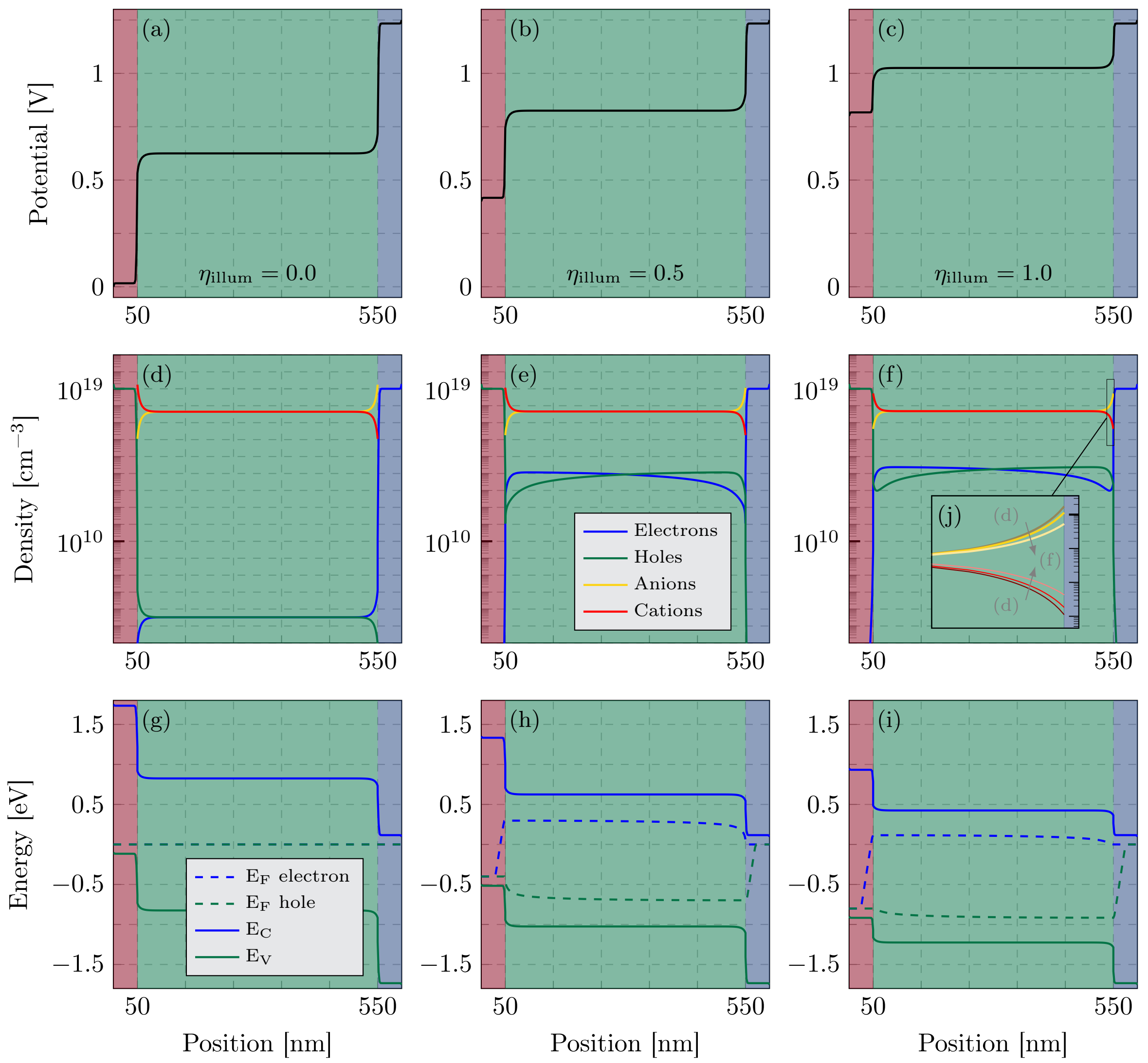

Figure S2: (a)-(c) Electrostatic potential, (d)-(f) charge carrier density, and (g)-(i) quasi Fermi energy, conduction band minimum, and valence band maximum profiles in the dark and at $\eta_{\text{illum}} = 0.5$ and 1.0 respectively. (j) Zoom on the ion accumulation at the interface as shown in figures (d)-(f). The arrows mark the direction of increasing illumination intensity. The electric field screening by the ions is visible as a constant electrostatic potential throughout most of the perovskite layer. The illumination-dependent generation of electronic charge carriers leads to an increase in their density throughout the device and to the splitting of the quasi Fermi energies of the electrons and holes. Note that, despite all simulations being performed at short-circuit, the boundary potential changes under illumination due to the influence of the series resistance.

Alternating potential profiles in the dark

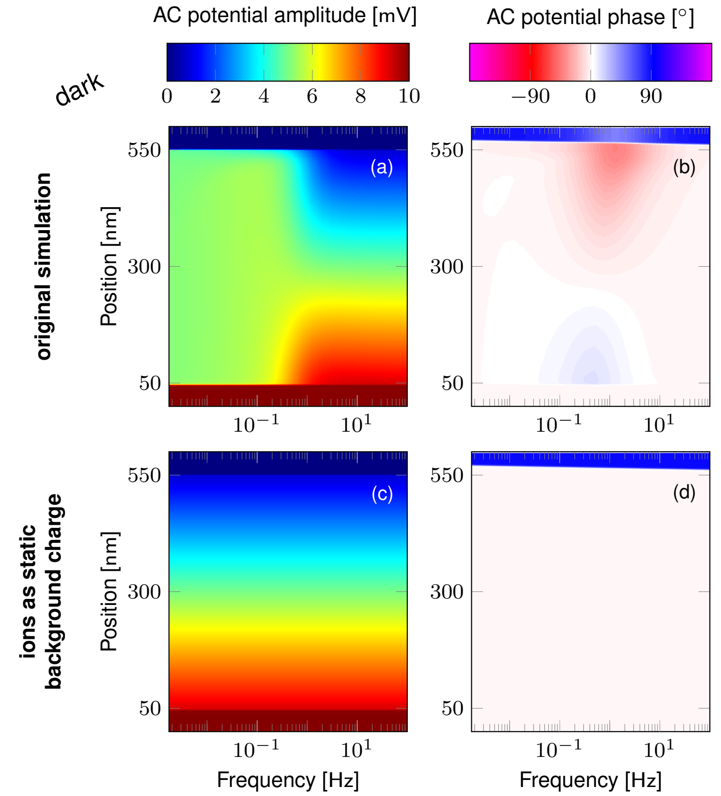

Figure S3: (a),(c) Amplitude and (b),(d) phase of the alternating potential in the dark of (a)-(b) the original simulation and (c)-(d) the simulation treating the ions as static background charge. The alternating electric field below 1 Hz is screened by the oscillating ions. While this is analogous to the results under illumination shown in figure 3, the influence on the overall results is significantly smaller due to the smaller concentration of electronic charge carriers.

Choice of capacitance at which to extract the frequency

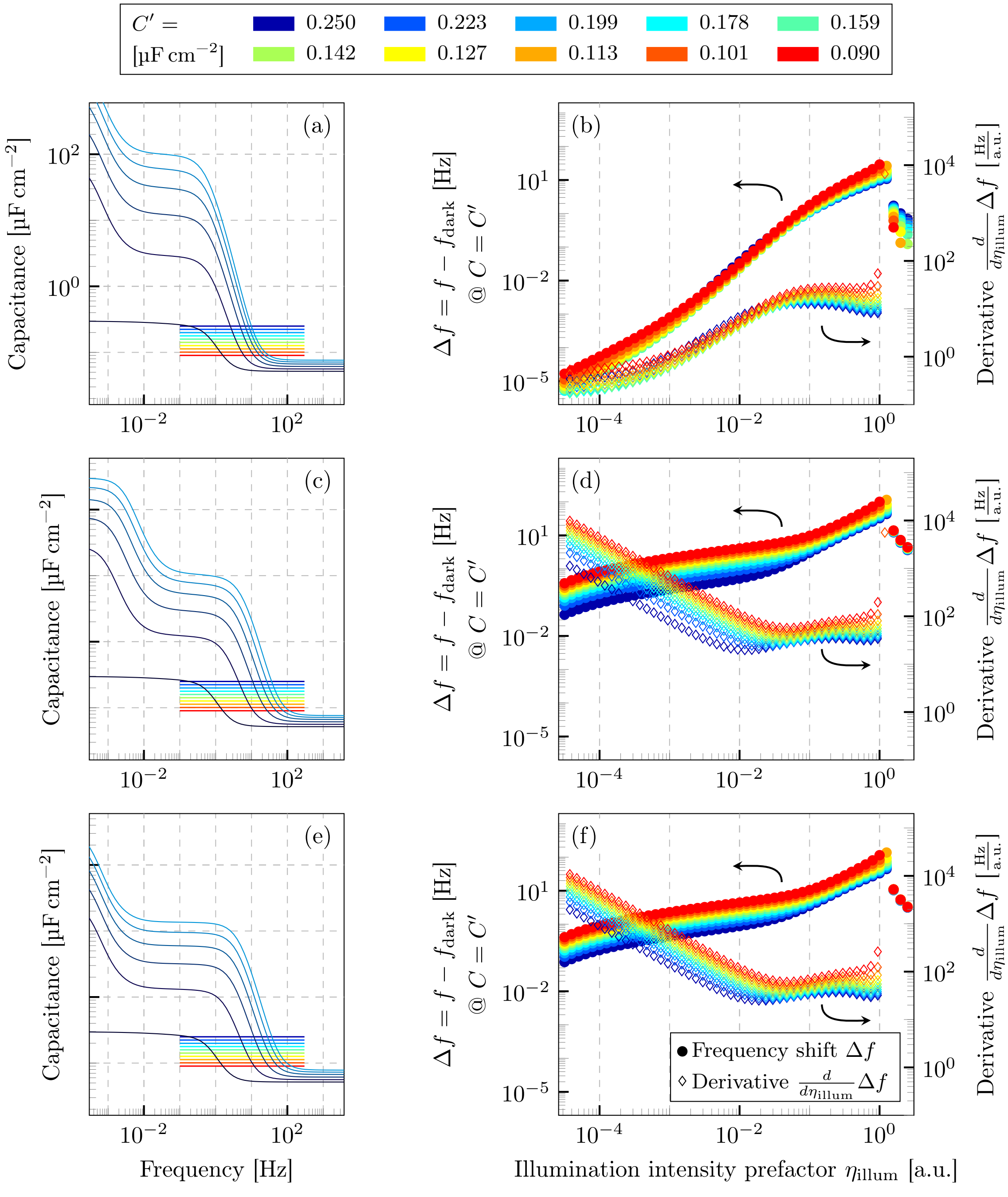

Figure S4: Capacitance over frequency and frequency shift and its derivative over illumination intensity at different capacitance values C' obtained for (a)-(b) the original simulation and the same simulation but additionally increasing the (c)-(d) ion mobility respectively (e)-(f) ion concentration in parallel to the illumination. The qualitative shape of the frequency shift and its derivative are independent from the exact choice of the capacitance value C'.

Negative capacitance at high illumination intensity

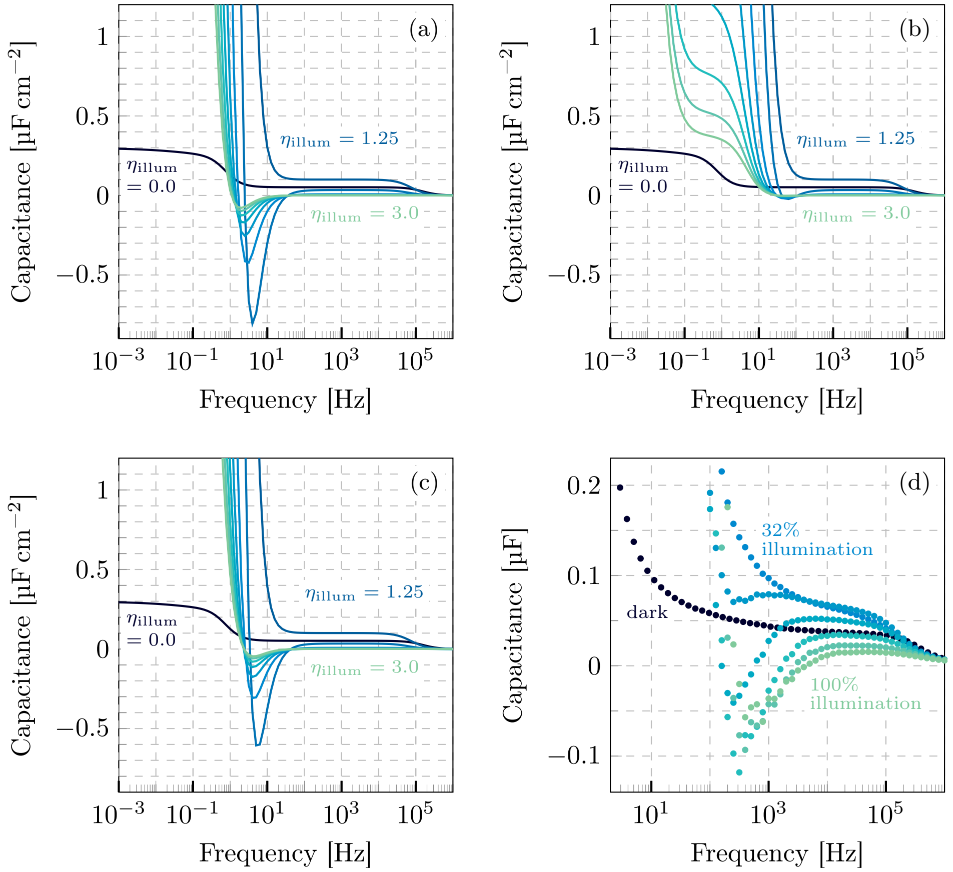

Figure S5: Illumination-dependent capacitance at high illumination intensities (a) if only the illumination intensity is varied and (b) if the ion mobilities respectively (c) the ion densities are increased in parallel to the illumination intensity. (d) Illumination-dependent capacitance at high illumination intensities measured on a PSC with a carbon-based back-electrode (the same as in figures 5 (a) and figure S6 (a)). The illumination intensity at which the continuous frequency shift ends and the capacitance might become negative can be used as a point of reference for the analysis of the illumination intensity sweep.

Capacitance and conductance measurement results

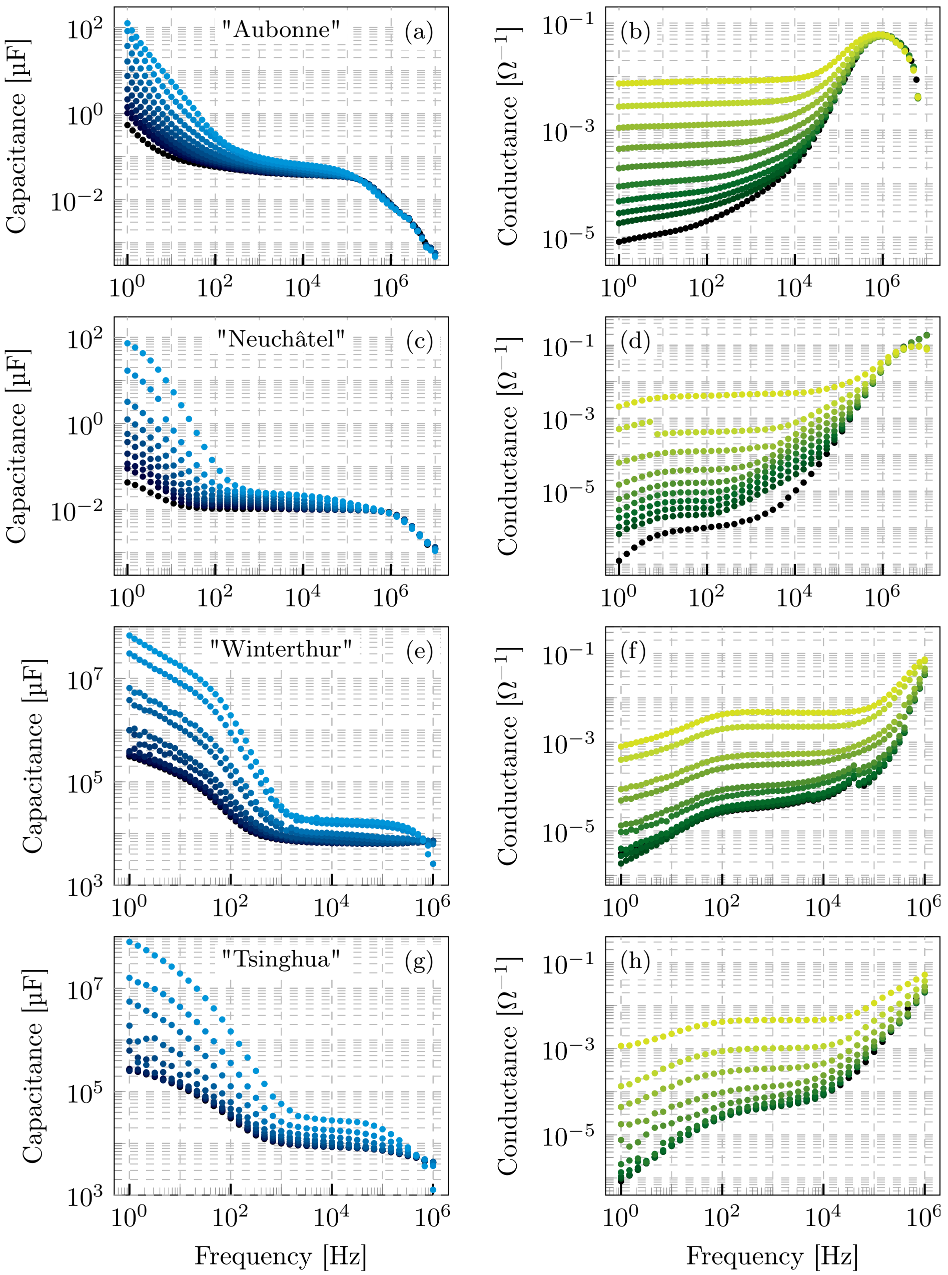

Figure S6: Illumination-dependent capacitance and conductance measurements for (a)-(b) a PSC with a carbon-based back-electrode, (c)-(d) a double cation PSC, (e)-(f) a triple cation PSC, and (g)-(h) a FAPbI PSC. These measurements provided the basis for the plots in figure 5. The black curve represents the measurement in the dark and the shade of blue and green, respectively, become brighter the higher the illumination intensity. Please note that the magnitude of the high-frequency capacitance plateau is increasing to a varied extent with increasing illumination intensity. This effect has been reported by Almora et al.[S8]. This can be seen as well in the simulation and does not affect the analysis of the frequency shift.

Influence of recombination

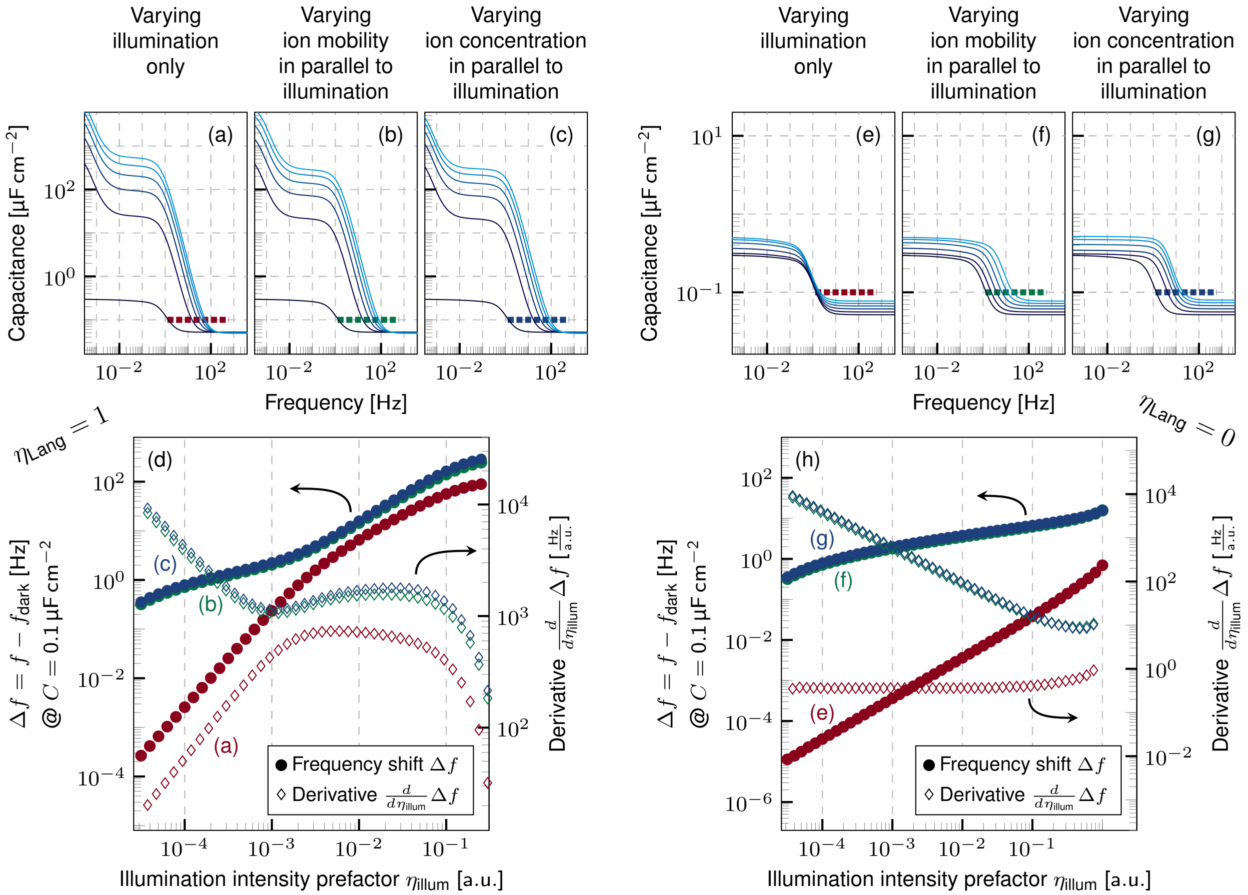

Figure S7: Results for simulations with (a)-(d) 3 orders of magnitude larger recombination efficiency in the perovskite layer ($\eta_{\text{Lang}} = 1$) and (e)-(h) no recombination at all ($\eta_{\text{Lang}} = 0$). Capacitance obtained for (a),(e) the original simulation and the same simulation but additionally increasing the (b),(f) ion mobility or (c),(g) ion concentration, respectively, in parallel to the illumination. (d),(h) Frequency shift of the capacitance onset and its derivative for the three cases. The increased recombination strongly affects the results, but the method to assess a photo-conductive effect on the ion conductivity remains valid. The simulation without any recombination terms still shows the increase in capacitance in the low-frequency range but with decreased magnitude. This is an edge case, causing the derivative of the original simulation (e) to remain constant towards lower illumination intensities.

References

(S1) Setfos 5.5 User Manual. Fluxim AG, 2024.

(S2) Van Roosbroeck, W. W. Theory of the Flow of Electrons and Holes in Germanium and Other Semiconductors. *Bell System Technical Journal* **1950**, *29*, 560–607.

(S3) Langevin, P. Recombinaison et Mobilités des Ions dans les Gaz. *Annales de chimie et de physique* **1903**, *28*, 433–530.

(S4) van der Holst, J. J. M.; van Oost, F. W. A.; Coehoorn, R.; Bobbert, P. A. Electron-hole recombination in disordered organic semiconductors: Validity of the Langevin formula. *Physical Review B* **2009**, *80*, 235202.

(S5) Simlinger, T. Simulation von Heterostruktur-Feldeffekttransistoren. Ph.D. thesis, Technische Universität Wien, 1996.

(S6) Neukom, M. T.; Schiller, A.; Züfle, S.; Knapp, E.; Ávila, J.; Pérez-del-Rey, D.; Dreessen, C.; Zanoni, K. P. S.; Sessolo, M.; Bolink, H. J. et al. Consistent Device Simulation Model Describing Perovskite Solar Cells in Steady-State, Transient, and Frequency Domain. *ACS Applied Materials & Interfaces* **2019**, *11*, 23320–23328.

(S7) Laux, S. Techniques for Small-Signal Analysis of Semiconductor Devices. *IEEE Transactions on Computer-Aided Design of Integrated Circuits and Systems* **1985**, *4*, 472–481.

(S8) Almora, O.; Aranda, C.; Garcia-Belmonte, G. Do Capacitance Measurements Reveal Light-Induced Bulk Dielectric Changes in Photovoltaic Perovskites? *The Journal of Physical Chemistry C* **2018**, *122*, 13450–13454.
