# Supplementary material for: Assessing the Influence of Illumination on Ion Conductivity in Perovskite Solar Cells
Source: J Phys Chem Lett. 2024 Nov 3;15(45):11252–8. doi: 10.1021/acs.jpclett.4c02403 (PMC11571209; doi:10.1021/acs.jpclett.4c02403)
Supplement: Supplementary file 2 — jz4c02403_si_002.pdf [file jz4c02403_si_002.pdf]

jz-2024-02403u.R1

Name: Peer Review Information for "Assessing the Influence of Illumination on Ion Conductivity in Perovskite Solar Cells"

First Round of Reviewer Comments

Reviewer: 1

Comments to the Author

The authors present an interesting study of an important effect widely observed in perovskite solar cells. They show a compelling mechanism for explaining an interaction of ac modulated charge that causes enhanced capacitance under illumination.

However there are some points to improve in order to show that this mechanism actually explains the physical behaviour of perovskite solar cells.

1. Eqs. (1) are not clear. The authors should check that these equations are true. In the measurement one measures the impedance and the capacitance and conductance are defined posteriorly by standard formulas. I do not know if one can define capacitance and conductance separately. Please check.

2. The model is too simplistic. Clearly this is a model from OLEDs that operate in high injection. Clearly the boundary conditions shown in SI are not valid in a solar cell. This cast doubt on the interpretation. The work of A. Walker and coworkers has shown that the boundary conditions are essential because the ion polarization mainly occurs ate the interface (Richardson, G.; O'Kane, S. E. J.; Niemann, R. G.; Peltola, T. A.; Foster, J. M.; Cameron, P. J.; Walker, A. B. Can slow-moving ions explain hysteresis in the current-voltage curves of perovskite solar cells?, Energy Environ. Sci. 2016, 9, 1476-1485.). That’s why transport layers are mandatory in the recent drift-diffusion studies.

3. The low frequency capacitance of perovskite solar cells is found experimentally to be very sensitive to the contact properties. Sometimes the light induced capacitance even disappears, especially with organic contacts. The present work shows a purely bulk effect, associated to modulated of bulk ionic charge, as in OLEDs. This is really not clear from the presentation. The authors could plot carrier distributions to better show what they are meaning.

4. Coupling of the electronic charge with the ionic charge has been commented many times. It would be useful if the authors connect the discussion to other phenomena that have been widely studied, for example the negative capacitance. For example the following works suggested a charge coupling at the interface, with the slowing down by ions producing some electron dynamic properties: Kovalenko, A.; Pospisil, J.; Krajcovic, J.; Weiter, M.; Guerrero, A.; Garcia-Belmonte, G. Interface inductive currents and carrier injection in hybrid perovskite single crystals, App. Phys.

Lett. 2017, 111, 163504; Ghahremanirad, E.; Bou, A.; Olyaee, S.; Bisquert, J. Inductive Loop in the Impedance Response of Perovskite Solar Cells Explained by Surface Polarization Model, J. Phys. Chem. Lett. 2017, 8, 1402-1406. But other works have proposed a similar coupling mechanism by the general electrical field in the bulk: Wu, J.; Yang, C.; Luo, Z.; Wang, X.; Zheng, F.; Zhao, Z.; Hu, Z. Unified model for describing the evolution of negative capacitance in perovskite solar cells, Phys. Rev. Appl. 2024, 22, 024041.

5. The authors present a valuable study of the frequency of onset of the capacitance. However the solar cells are very different, and here linked a priori with the ion conductance (that is not measured). Please show also the conductance plots to see any possible correlation.

6. It should be commented that in some cells (c) the high frequency plateau depends on light intensity. (Almora, O.; Aranda, C.; Garcia-Belmonte, G. Do Capacitance Measurements Reveal Light-Induced Bulk Dielectric Changes in Photovoltaic Perovskites?, J. Phys. Chem. C 2018, 122, 13450-13454.)

7. Recombination is essential to solar cells. All injected current is recombination current. At Voc the internal recombination current balances the generation. I cannot understand in which sense the authors mean “removing recombination”.

In summary, the work shows an important and well-argued mechanism on light-induced capacitance. I would like to see a broader discussion, for example connecting these results with negative capacitance and Voc decay properties. The authors suggest this is the governing mechanism in perovskite solar cells, and not just a speculation. I proposed further experimental evidence with varied devices of a single active material should be presented, and the results correlated with some internal parameters.

Reviewer: 2

Comments to the Author

This paper provides a useful discussion of the effects of illumination on ionic conductivity. The approach to deducing capacitance shown in eqs 1a and 1b is a valid alternative to using the complex impedance, see Clarke et al 2024 DOI 10.1002/aenm.202400955, and would have advantages over equivalent circuits as pointed out by Clarke et al for their own approach. Its novelty and interest to physical chemists, not just specialists working in the authors' area of research is difficult to judge. For example, it should be clear about which consequences of ionic motion under an alternating field described in this paper are not already described in published work. Specific concerns that need to be addressed before this paper can be considered for publication are listed below. 1. On page 3 the paper refers to 'the assumption of independent electronic and ionic charge

transport' In what sense are electronic and ionic charge transport independent? Does Langevin recombination (SI P2) lead to significantly different predictions from the standard Shockley-Read-Hall expression with appropriate parameters? As the authors point out, Langevin recombination is often used to model organic semiconductors where charge transport is by hopping, not the case for perovskites. This difference in recombination models makes it hard to compare the results presented here with results presented elsewhere in this widely studied topic. 2. Page 3 states 'This investigation starts with triple cation PSCs using the stack described by Yavari et al.23.' The device architecture should be stated and the SI P4 figure moved here. 3. Pages 4-5 'As mentioned, the origin of the effects at low frequencies was the topic of debate. The frequency range suggests a contribution by the slow ionic charge carriers while the sensitivity to illumination hints toward an electronic effect.' These sentences need clarification. 4. Pages 4-5 The statement 'Additional effects such as an illumination- dependent dielectric constant, activation energy, or increase in defects can be excluded as they are not covered by the model used to reproduce the measurement results' implies that the additional effects influencing impedance would not be seen in the measurements, and so are not causes of the changes in impedance. Why that is the case should be demonstrated. 5. P 8 it states 'An elegant approach suggested here is to include the charge carriers in the steady-state simulation and treat their distribution as a static background charge, like e.g. a doping density, within the AC simulation'. The strategy of identifying the importance of the ions being mobile by comparing with results when fixing the ions in place raises the question of what profile for the static ions should be used. The authors should show what the ion profile is and how it is determined given the profile will depend significantly on the bias history of the device as pointed out by many authors. It is therefore difficult to understand the argument that illumination-dependent effects vanish if the ions are kept static. 6. SI Figure S1: all parameter values should be cited.

Author's Response to Peer Review Comments:

Senior Editor
The Journal of Physical Chemistry Letters
ACS Publications
1155 Sixteenth Street N.W.
Washington, DC 20036

Fluxim AG
Dr. Andreas Schiller
Katharina-Sulzer-Platz 2
CH-8400 Winterthur
Switzerland
Phone: +41 44 500 4770
Email: andreas.schiller@fluxim.com

Winterthur, 7th September 2024

Reply to revision

Dear Editor

Thank you for the swift reply to our contribution "Assessing the Influence of Illumination on Ion Conductivity in Perovskite Solar Cells" to the special issue in the *Journal of Physical Chemistry Letters*.

First of all, I have to admit, that we discovered that some data was mislabelled when transferred from ZHAW to Fluxim. Thus, the data for the devices "Winterthur" and "Tsinghua" was incorrect in the submitted version. We've now carefully reviewed the data and corrected the respective plots in figures 5 and S5. This does not affect the findings or conclusion of the work. I sincerely apologize for my mistake.

We'd like to thank the two reviewers for their comments and questions, which we will address in the following.

Reviewer 1:

1) `Eqs. (1) are not clear. The authors should check that these equations are true. In the measurement one measures the impedance and the capacitance and conductance are defined posteriorly by standard formulas. I do not know if one can define capacitance and conductance separately. Please check.`

   The impedance is computed as $Z = \frac{V_{AC}}{I_{AC}}$, where the complex quantities V_{AC} and I_{AC} are computed from $v(t) = V_{steady-state} + Re(V_{AC} \cdot e^{i\omega t})$ and $i(t) = I_{steady-state} + Re(I_{AC} \cdot e^{i\omega t})$. Conductance and capacitance are then derived from the impedance as $G = Re\left(\frac{1}{Z}\right)$ and $C = \frac{1}{\omega} \cdot Im\left(\frac{1}{Z}\right)$. In simulation, we choose $V_{AC} = V_{amp}$ to be real (i.e. the applied voltage has no phase shift, this happens without any loss of generality). As the total current density is constant throughout the device, it can be evaluated at any arbitrary point in the device or – as in equations (1a) and (1b) – computed by taking the average $I_{AC}(f) = \frac{\int I_{AC}(f,x)dx}{\int dx}$. Since the current at each position within the device is a superposition of the charge carrier currents and the displacement current $I_{AC}(f,x) = I_n(f,x) + I_p(f,x) + I_a(f,x) + I_c(f,x) + I_{disp}(f,x)$, the integral can be written inside the sum. Substituting the integral into the equation for impedance and the impedance in the equation for conductance and capacitance results in equations (1a) and (1b). In measurement, the components can of course not be distinguished, but in simulation, the current component profiles are available.

2) The model is too simplistic. Clearly this is a model from OLEDs that operate in high injection. Clearly the boundary conditions shown in SI are not valid in a solar cell. This cast doubt on the interpretation. The work of A. Walker and coworkers has shown that the boundary conditions are essential because the ion polarization mainly occurs ate the interface (Richardson, G.; O'Kane, S. E. J.; Niemann, R. G.; Peltola, T. A.; Foster, J. M.; Cameron, P. J.; Walker, A. B. Can slow-moving ions explain hysteresis in the current-voltage curves of perovskite solar cells?, Energy Environ. Sci. 2016, 9, 1476-1485.). That's why transport layers are mandatory in the recent drift-diffusion studies.

The model is meant to be as complex as necessary but as simple as possible, to show that the overall effects can be reproduced solely by electronic-ionic interaction, and do not depend on any more advanced models. We are using the same boundary conditions as used by G. Richardson et al. Additionally, we include the transport layers in our device stack. In agreement with their assumptions, our transport layers are highly doped for efficient charge extraction. Our model, including the boundary conditions, has been validated in previous work. [M.T. Neukom, A. Schiller, S. Züfle, E. Knapp, J. Àvila, D. Pérez-del-Rey, C. Dreessen, K.P.S. Zanoni, M. Sessolo, H.J. Bolink, B. Ruhstaller, Consistent Device Simulation Model Describing Perovskite Solar Cells in Steady-State, Transient, and Frequency Domain, ACS Appl. Mater. Interfaces, 2019, 11, 26, 23320-23328]

3) The low frequency capacitance of perovskite solar cells is found experimentally to be very sensitive to the contact properties. Sometimes the light induced capacitance even disappears, especially with organic contacts. The present work shows a purely bulk effect, associated to modulated of bulk ionic charge, as in OLEDs. This is really not clear from the presentation. The authors could plot carrier distributions to better show what they are meaning.

While the effect mainly emerges in the bulk of the perovskite layer, it is also influenced by the contact parameters via the concentration of electronic charge carriers. We've added a figure (S2) in the supporting information showing the illumination-dependent steady-state profiles including the charge carrier profiles. It shows the substantial increase in the density of electronic charge carriers under illumination which is one of the causes for the low-frequency effects.

We've always observed an illumination-induced increase in the low-frequency capacitance in mixed electronic-ionic conductors and we are not aware of any contrary reports in literature.

4) Coupling of the electronic charge with the ionic charge has been commented many times. It would be useful if the authors connect the discussion to other phenomena that have been widely studied, for example the negative capacitance. For example the following works suggested a charge coupling at the interface, with the slowing down by ions producing some electron dynamic properties: Kovalenko, A.; Pospisil, J.; Krajcovic, J.; Weiter, M.; Guerrero, A.; Garcia-Belmonte, G. Interface inductive currents and carrier injection in hybrid perovskite single crystals, App. Phys. Lett. 2017, 111, 163504; Ghahremanirad, E.; Bou, A.; Olyaee, S.; Bisquert, J. Inductive Loop in the Impedance Response of Perovskite Solar Cells Explained by Surface Polarization Model, J. Phys. Chem. Lett. 2017, 8, 1402-1406. But other works have proposed a similar coupling mechanism by the general electrical field in the bulk: Wu, J.; Yang, C.; Luo, Z.; Wang, X.; Zheng, F.; Zhao, Z.; Hu, Z. Unified model for describing the evolution of negative capacitance in perovskite solar cells, Phys. Rev. Appl. 2024, 22, 024041.

Similar to the findings presented here, negative capacitance can be modeled by solely considering electronic-ionic interaction. A very complete study has recently been released by *W. Clarke, G. Richardson, P. Cameron, Understanding the Full Zoo of Perovskite Solar Cell Impedance Spectra with the Standard Drift-Diffusion Model. Adv. Energy Mater. 2024, 14, 2400955.* using a drift-diffusion model very similar to ours and several different device structures. We reformulated the sentence to specifically name EIS features reproduced by drift-diffusion simulations and included the citations for J. Wu et al. and W. Clarke et al. which are based on drift-diffusion models.

Also, drift-diffusion simulations of EIS that complement the established models by mixed electronic-ionic interaction are well established to qualitatively and quantitatively reproduce measurement results such as multiple arcs in the Cole-Cole diagram of the impedance, an increase in capacitance at low frequencies, and negative capacitance [13,18–24].
While the methods presented here could most certainly help the understanding of the origin of these effects, we believe it would exceed the scope of this work, whose focus lies on the photoconductive effect of ionic charges.

5) The authors present a valuable study of the frequency of onset of the capacitance. However the solar cells are very different, and here linked a priori with the ion conductance (that is not measured). Please show also the conductance plots to see any possible correlation.
We've included the conductance plots in figure S5 in the supporting information.

6) It should be commented that in some cells (c) the high frequency plateau depends on light intensity. (Almora, O.; Aranda, C.; Garcia-Belmonte, G. Do Capacitance Measurements Reveal Light-Induced Bulk Dielectric Changes in Photovoltaic Perovskites?, J. Phys. Chem. C 2018, 122, 13450-13454.)
Indeed, all the cells show an increase in capacitance with increasing illumination intensity to a varied extent. We have mentioned this increase in magnitude of the high-frequency plateau in the caption of figure S5 and referenced the corresponding publication. Please note that this does not influence the analysis of the frequency shift.

7) Recombination is essential to solar cells. All injected current is recombination current. At Voc the internal recombination current balances the generation. I cannot understand in which sense the authors mean "removing recombination".
First, we would like to point out that the impedance was measured under short-circuit conditions. At this operation point, the majority of charge carriers are extracted.
We agree, that it is not possible to fabricate a device without recombination. In simulation, however, we can disable the respective terms without affecting other mechanisms such as optical generation, injection, or extraction. As in this case, the effects persist, we rule out recombination as the leading cause for the phase shift.
We realized that we only mention the short-circuit condition once and thus added it on two additional occasions in the manuscript.

In summary, the work shows an important and well-argued mechanism on light-induced capacitance. I would like to see a broader discussion, for example connecting these results with negative capacitance and Voc decay properties. The authors suggest this is the governing mechanism in perovskite solar cells, and not just a speculation. I proposed further experimental evidence with varied devices of a single active material should be presented, and the results correlated with some internal parameters.
We completely agree that further experimental and theoretical work is necessary to improve the understanding of photoactivation mechanisms in PSCs. However, such a comprehensive study analyzing the effect of photoconductivity on working points would go beyond the scope of a single publication.
Our model is based on well-established physical properties (mixed electronic-ionic conduction) or is modeling an effect that has been proclaimed in literature (photo-conductive effect on ions). We thus do not speculate about new governing mechanisms, but show how the established models reproduce EIS measurements and allow to assess the presence of a photo-conductive effect.

Reviewer 2:

1) On page 3 the paper refers to 'the assumption of independent electronic and ionic charge transport' In what sense are electronic and ionic charge transport independent? Does Langevin recombination (SI P2) lead to significantly different predictions from the standard Shockley-Read-Hall expression with appropriate parameters? As the authors point out, Langevin recombination is often used to model organic semiconductors where charge transport is by hopping, not the case for perovskites. This difference in recombination models makes it hard to compare the results presented here with results presented elsewhere in this widely studied topic.

   The concept of independent electronic and ionic charge transport is applied in the cited reference [5]. We mention this, as we'd have to include a light-induced reduction of the activation energy of ions if we'd agree with the findings of reference [5].

   Langevin recombination is equivalent to bimolecular recombination with a respective prefactor (we've added a respective comment in the supporting information). It is used exclusively here to keep the model simple and avoid additional parameters (trap depth, density, and capture rates). Using SRH recombination leads to quantitatively different results, but the discussed qualitative behavior is preserved.

2) Page 3 states 'This investigation starts with triple cation PSCs using the stack described by Yavari et al.23.' The device architecture should be stated and the SI P4 figure moved here.

   We've included the device stack in the text. As the author's guidelines restrict a letter to 3-5 figures, we had to prioritize. The measurements in figure S5 were only conducted to extract the frequency shift and have thus a limited range towards lower frequencies.

3) Pages 4-5 'As mentioned, the origin of the effects at low frequencies was the topic of debate. The frequency range suggests a contribution by the slow ionic charge carriers while the sensitivity to illumination hints toward an electronic effect.' These sentences need clarification.

   We've added explanations and a reference to the two sentences mentioned:

   As mentioned in the first two paragraphs, the origin of the characteristic low-frequency response has been the topic of debate. The frequency range of the capacity increase suggests a contribution by the slow ionic charge carriers [14], while its sensitivity to illumination hints toward an electronic mechanism, as in the model, only the electronic charge carriers are directly linked to the illumination intensity via the optical generation.

4) Pages 4-5 The statement 'Additional effects such as an illumination-dependent dielectric constant, activation energy, or increase in defects can be excluded as they are not covered by the model used to reproduce the measurement results' implies that the additional effects influencing impedance would not be seen in the measurements, and so are not causes of the changes in impedance. Why that is the case should be demonstrated.

   The mentioned additional effects can be ruled out as the cause for the effects in simulation, as they are not part of the model. We've clarified that in the respective sentence.

   Additional effects such as an illumination-dependent dielectric constant, activation energy, or increase in defects can be ruled out as cause for the illumination-dependent capacitance in the simulation, since they are not included in the present model used to reproduce the measurement results.

   Surely, the model could be extended to include these additional effects. However, their use in a model would need additional justification. Important in this work is that they are not necessary to reproduce the measurements.

5) P 8 it states 'An elegant approach suggested here is to include the charge carriers in the steady-state simulation and treat their distribution as a static background charge, like e.g. a doping density, within the AC simulation'. The strategy of identifying the importance of the ions being

mobile by comparing with results when fixing the ions in place raises the question of what profile for the static ions should be used. The authors should show what the ion profile is and how it is determined given the profile will depend significantly on the bias history of the device as pointed out by many authors. It is therefore difficult to understand the argument that illumination-dependent effects vanish if the ions are kept static.

We're using the respective steady-state ion distribution at the selected illumination intensity and at short-circuit. The steady-state solution needs to be computed as the basis for any impedance simulation and is solved at the respective illumination intensity. We've refined the preceding sentence to improve the comprehensibility of the method.

The effects vanish because they are not directly caused by illumination but by the increased concentration of electronic charge carriers combined with the screening of the AC electric field by the oscillating ions. Keeping the oscillating ions static results in an increase in conductivity (see dash-dotted lines in figure 3(b)) due to the increased concentration of electronic charge carriers induced by the increased optical generation. However, without the screening of the AC electric field, the results lack the decrease in conductivity and increase in capacitance below 10 Hz.

6) SI Figure S1: all parameter values should be cited.

We've mentioned the origin of the order of magnitude of the parameters. However, the model is intentionally kept as simple as possible and thus many parameters have been modified for the sake of simplicity and/or symmetry.

We've also incorporated late feedback from a co-author regarding phrasing and language. Furthermore, we implemented the requested non-scientific changes and made use of the additional space in the TOC graphic.

All changes are marked in the annexed version for review of both the manuscript and the supporting information.

Yours sincerely,

Andreas Schiller

jz-2024-02403u.R2

Name: Peer Review Information for "Assessing the Influence of Illumination on Ion Conductivity in Perovskite Solar Cells"

Second Round of Reviewer Comments

Reviewer: 2

Comments to the Author

The authors have fully answered the reviewers' comments and the revised paper can be accepted for publication in JPCL.

Reviewer: 3

Comments to the Author

Manuscript: “Assessing the Influence of Illumination on Ion Conductivity in Perovskite Solar Cells”

The authors present an interesting and useful work focus on the effects of illumination on ionic conductivity. To do that, they interpret the illumination-dependent low frequency behaviour in impedance spectroscopy IS by drift-diffusion DD simulations. They propose a characterization technique to detect photo-induced increase in ionic conductivity based on the shift of the onset of the capacitance with the light. It is a well-argued work. However, specific questions need to be addressed before this paper will be published.

1) I suggest including the derivation of the equation 1a and 1b in the SI.

2) Figure S2: Are (h) and (i) the energy diagrams at open circuit? I suggest plotting them at short-circuit as it is the condition in which the authors have performed all the simulations. In addition, it will be interesting to show if there is or there is not a variation on the ion distribution with the light intensity plotting in another figure the superposition to all of them.

3) Figure 3 is full of interesting and useful results. Figures 3 (c)-(f), are simulated at a specific time during the “measurement of the impedance”, right? Therefore, if we take other time, do the capacitive and inductance performance in (d) and (f) change?

4) I agree with Referee 1 that recombination is essential to solar cells, and it is not possible to remove it. From drift-diffusion simulations, the magnitude of the onset frequency can depend on the recombination rate according from the simulations done on the following paper

https://doi.org/10.1063/5.0216983. I think that is too strong to claim in the text: “without considering any recombination mechanisms.” As soon as you are closer to higher charge concentrations, this recombination could be more significative as it is happening at higher light intensities in which you can find even negative capacitances. Then, as the authors claim, another point it is the impact on the shift of the onset. Therefore, I advise running Figure 4 with a significative higher recombination rate to check if there is no impact on the shift (or what maybe it is more important, with respect to the sign of the slope of the derivative of the shift).

FORMAT--

5/ Please, check the format of the references. In some of them, there is not the name of the journal.

6/ Only for Figure S4, please, change the colours of the curves because they look quite similar for the different illuminations.

Reviewer: 1

Comments to the Author

The standard definition of the frequency dependent capacitance in terms of the impedance is C(w)=1/iwZ(w). The same method is applied for the frequency dependent dielectric constant in relation to the complex conductivity. Then you take real and imaginary parts. It is not clear if the Eqs (1) and (2) satisfy this relation, as the capacitance and conductance are calculated from the current. Please clarify the meaning, of the C and G that you are introducing.

The authors should try to give a satisfactory response to the question of surface vs. bulk effects. This is a key question realted to the appearance of negative capacitances that has been investigated experimentally (Aranda, C. A.; Alvarez, A. O.; Chivrony, V. S.; Das, C.; Rai, M.; Saliba, M. Overcoming ionic migration in perovskite solar cells through alkali metals, Joule 2024, 8, 241-254.) In the simulations the electronic charge is accumulated at the surface while the model does not simulate realistic surface charge. Maybe the authors can double the cell thickness at the same photocurrent to see the scaling of the capacitance, or a similar procedure informative to readers, that may check this model with experiments.

Author's Response to Peer Review Comments:

Senior Editor
The Journal of Physical Chemistry Letters
ACS Publications
1155 Sixteenth Street N.W.
Washington, DC 20036

Fluxim AG
Dr. Andreas Schiller
Katharina-Sulzer-Platz 2
CH-8400 Winterthur
Switzerland
Phone: +41 44 500 4770
Email: andreas.schiller@fluxim.com

Winterthur, 20th September 2024

Reply to revision

Dear Editor

Thank you for your reply regarding our contribution "Assessing the Influence of Illumination on Ion Conductivity in Perovskite Solar Cells" to the special issue in the *Journal of Physical Chemistry Letters*. We thank the reviewers for their valuable comments and questions, which we answer in the following.

Reviewer 3:

1) `I suggest including the derivation of the equation 1a and 1b in the SI.`
   As the origins of these equations cause more confusion than anticipated, we have added a respective section to the SI.

2) `Figure S2: Are (h) and (i) the energy diagrams at open circuit? I suggest plotting them at short-circuit as it is the condition in which the authors have performed all the simulations. In addition, it will be interesting to show if there is or there is not a variation on the ion distribution with the light intensity plotting in another figure the superposition to all of them.`
   All simulations are performed at short-circuit. The influence of the series resistance causes a shift in the boundary potential which might appear as if the applied voltage was changed. We have added a clarifying statement to the caption of the figure.
   The ion profiles change slightly under illumination due to the slightly smaller potential gradient or electric field, respectively. We have added an inset to figure S2 zooming into the ion accumulations at the interface and combining the ion densities of all three simulations.

3) `Figure 3 is full of interesting and useful results. Figures 3 (c)-(f), are simulated at a specific time during the "measurement of the impedance", right? Therefore, if we take other time, do the capacitive and inductance performance in (d) and (f) change?`
   Figures 3 (c)-(f) show amplitude $|\psi_{AC}|$ and phase $\angle\psi_{AC}$ of the complex AC potential. The potential at a specific time would be computed as $\psi(x,t) = \psi_0(x) + \Re(\psi_{AC}(x) \cdot e^{i\omega t}) = \psi_0(x) + |\psi_{AC}(x)| \cdot \Re(e^{i\omega t + \angle\psi_{AC}(x)})$.

4) I agree with Referee 1 that recombination is essential to solar cells, and it is not possible to remove it. From drift-diffusion simulations, the magnitude of the onset frequency can depend on the recombination rate according from the simulations done on the following paper https://doi.org/10.1063/5.0216983. I think that is too strong to claim in the text: "without considering any recombination mechanisms." As soon as you are closer to higher charge concentrations, this recombination could be more significative as it is happening at higher light intensities in which you can find even negative capacitances. Then, as the authors claim, another point it is the impact on the shift of the onset. Therefore, I advise running Figure 4 with a significative higher recombination rate to check if there is no impact on the shift (or what maybe it is more important, with respect to the sign of the slope of the derivative of the shift).

We agree with both reviewers regarding the influence of the recombination on solar cells. However, in simulation we can set the bimolecular recombination terms to zero without affecting other processes. Due to the constant generation, the electrons and holes are then only coupled via the potential (and subsequently the electric field). In this simulation, we do not aim to simulate a realistic solar cell but only strive to investigate the influence of the bimolecular recombination terms in the model. Since the low-frequency capacitance and conductance effects under investigation do not vanish under these conditions, we exclude the recombination as the leading cause for the low-frequency capacitance and conductance effects under investigation.

For the sake of completeness, we've repeated the simulations for the two extreme situations of (1) a 3 orders of magnitude larger recombination efficiency and (2) a recombination efficiency of zero. The contents of figure 4 for these two cases have been added to the SI in figure S6. The overall results are not affected by the magnitude of the recombination.

5) Please, check the format of the references. In some of them, there is not the name of the journal.

Thanks for pointing that out. The style used in the template seems to not support certain bibtex keywords.

6) Only for Figure S4, please, change the colours of the curves because they look quite similar for the different illuminations.

We have extended the color scale into the light green to make the colors more distinguishable.

Reviewer 1:

The standard definition of the frequency dependent capacitance in terms of the impedance is C(w)=1/iwZ(w). The same method is applied for the frequency dependent dielectric constant in relation to the complex conductivity. Then you take real and imaginary parts. It is not clear if the Eqs (1) and (2) satisfy this relation, as the capacitance and conductance are calculated from the current. Please clarify the meaning, of the C and G that you are introducing.

We are using the parallel capacitance definition $C(\omega) = \frac{1}{\omega}\Im(Y(\omega)) = \frac{1}{\omega}\Im\left(\frac{1}{Z(\omega)}\right)$, which is – to our knowledge – more common in the field of semiconductor device physics. [Laux, 1985, doi.org/10.1109/tcad.1985.1270145], [Bonanos, Pissis, Macdonald, 2012,doi.org/10.1002/0471266965.com121]

We have specified the use of the parallel capacitance and conductance in the manuscript and added a section to the SI outlining the derivation of equations 1a and 1b including the definition of the parallel capacitance and conductance.

The authors should try to give a satisfactory response to the question of surface vs. bulk effects. This is a key question realted to the appearance of negative capacitances that has been investigated experimentally (Aranda, C. A.; Alvarez, A. O.; Chivrony, V. S.; Das, C.; Rai, M.; Saliba, M. Overcoming ionic migration in perovskite solar cells through alkali metals, Joule 2024, 8, 241-254.) In the simulations the electronic charge is accumulated at the surface while the model does not simulate realistic surface charge. Maybe the authors can double the cell thickness at the same photocurrent to see the scaling of the capacitance, or a similar procedure informative to readers, that may check this model with experiments.

We agree that the posed question is key to a better understanding of both impedance results of perovskite devices and their device physics. However, we don’t think that the device structure used for our simulations is suited for that kind of investigation as this would require a realistic set of parameters for expected surface effects and a proper consideration of the optical absorption profile. It further has to be considered that both the steady-state and the alternating electric field close to the interfaces strongly affect the demonstrated mechanisms. Thus, surface effects might also strongly influence the behavior of bulk effects and this might not be easily distinguishable from their direct influence on the impedance results.

All changes are marked in the annexed version for review of both the manuscript and the supporting information. The newly added sections in the SI are indicated by a highlighted section title.

Yours sincerely,

Andreas Schiller

jz-2024-02403u.R3

Name: Peer Review Information for "Assessing the Influence of Illumination on Ion Conductivity in Perovskite Solar Cells"

Third Round of Reviewer Comments

Reviewer: 1

Comments to the Author

I recommend publication

Reviewer: 3

Comments to the Author

Thank you for all your responses. I have few comments.

Previous question: Referee 3 : 2/ Figure S2: Are (h) and (i) the energy diagrams at open circuit?... // Authors: All simulations are performed at short-circuit. The influence of the series resistance causes a shift in the boundary potential which might appear as if the applied voltage was changed....

Referee 3: Thank you for your response. I don't fully understand Figure S2. From, figure S2b and S2c, the voltage drop across the series resistance for different illuminations are the same and approximately of 0.4V. However, from the difference of the QFL of the transport layers in figure S2h and S2i, we can expect a different voltage drop for different illuminations, which makes sense. Therefore, I am not sure if figure S2b and S2c are not the same figure. Please, could you check it?

It is interesting to remark that you are using a significant high series resistance which biases the solar cell when externally cero voltage is applied. This referee is curious of why you used 20ohm*cm2 for the series resistance (in any case, I don't think that is going to change your main message if you would have simulated with a smaller shunt resistance).

Previous question: Referee 3: 3/ Figure 3 is full of interesting and useful results…

Referee 3 : Thank you for your answer. I still think that Figure 3 is super important, and more information can be obtained. In Figure 3, it is interesting to see the different capacitive and inductive performance depending on the region of the device at a fixed frequency, although the device is quite symmetric. I suggest including the same figures for the case of dark and check the capacitive/inductive behaviours. Maybe, this new Figure can shed light, where does it come from the different capacitive/inductive performance of each region.

Previous question: Referee 3: 4) I agree with Referee 1 that recombination is essential to solar cells… // Authors: 4/ We agree with both reviewers regarding the influence of the recombination on solar cells. However, in simulation we can set the bimolecular recombination terms to zero without affecting other processes. Due to the constant generation, the electrons and holes are then only coupled via the potential (and subsequently the electric field). In this simulation, we do not aim to simulate a realistic solar cell but only strive to investigate the influence of the bimolecular recombination terms in the model. Since the low-frequency capacitance and conductance effects under investigation do not vanish under these conditions, we exclude the recombination as the leading cause for the low-frequency capacitance and conductance effects under investigation. For the sake of completeness, we've repeated the simulations for the two extreme situations of (1) a 3 orders of magnitude larger recombination efficiency and (2) a recombination efficiency of zero. The contents of figure 4 for these two cases have been added to the SI in figure S6. The overall results are not affected by the magnitude of the recombination.

Referee 3: I agree with you that the most essential mechanism which originates a high capacitance at low frequency is the electronic-ionic coupling as you are clearly presenting in Figure 2 in the manuscript. However, from my point of view, from your Figure S6, the recombination is substantially promoting and impacting on the magnitude of the capacitance and the frequency onset (https://doi.org/10.1063/5.0216983 in the SI Figure S43 and S46).

Influence of recombination

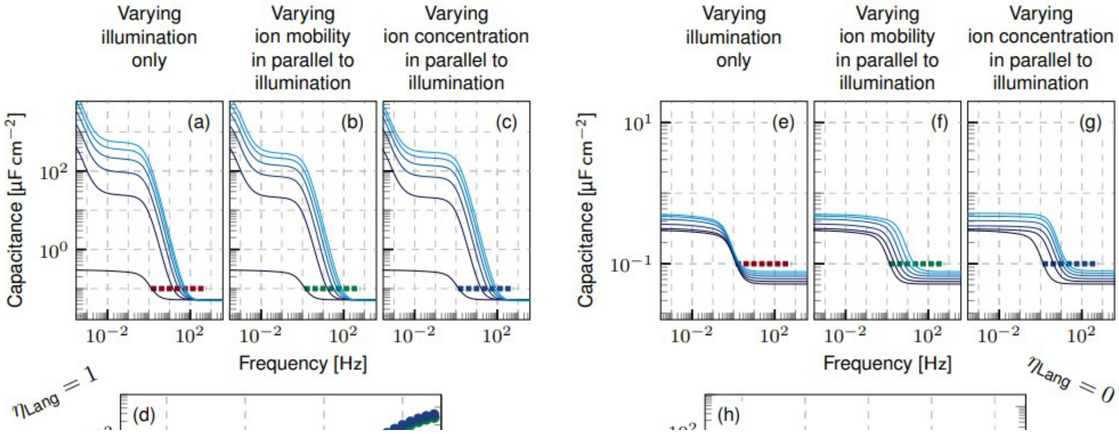

In the main text, you have written: "In contrast to previous studies, 12,13 the recombination, despite influencing the EIS results, is not causative for the large capacitance at low frequencies for the studied devices, as the effects persist even without considering any recombination mechanisms (figure S6(e) in the supporting information)." I am still thinking that is a strong statement to say, "effects persist even without considering any recombination mechanisms". From my point of view, the origin of the high capacitance at low frequency is the electronic-ionic coupling and its magnitude is promoted by the recombination.

Author's Response to Peer Review Comments:

Senior Editor
The Journal of Physical Chemistry Letters
ACS Publications
1155 Sixteenth Street N.W.
Washington, DC 20036

Fluxim AG
Dr. Andreas Schiller
Katharina-Sulzer-Platz 2
CH-8400 Winterthur
Switzerland
Phone: +41 44 500 4770
Email: andreas.schiller@fluxim.com

Winterthur, 2nd October 2024

Reply to revision

Dear Editor

Thank you for your reply regarding our contribution "Assessing the Influence of Illumination on Ion Conductivity in Perovskite Solar Cells" to the special issue in the *Journal of Physical Chemistry Letters*. In the following, we will address the reviewer's questions.

Reviewer 3:

`I don't fully understand Figure S2. From, figure S2b and S2c, the voltage drop across the series resistance for different illuminations are the same and approximately of 0.4V. However, from the difference of the QFL of the transport layers in figure S2h and S2i, we can expect a different voltage drop for different illuminations, which makes sense. Therefore, I am not sure if figure S2b and S2c are not the same figure. Please, could you check it?`

We have corrected the data of plot S2c. Thank you for pointing that out.

`It is interesting to remark that you are using a significant high series resistance which biases the solar cell when externally cero voltage is applied. This referee is curious of why you used 20ohm*cm2 for the series resistance (in any case, I don't think that is going to change your main message if you would have simulated with a smaller shunt resistance).`

The series resistance was at some point chosen to reproduce the roll-off of the capacitance at high frequencies (above 100 kHz). It was not questioned anymore as its magnitude does not significantly influence the qualitative properties of the simulation results at lower frequencies.

`Thank you for your answer. I still think that Figure 3 is super important, and more information can be obtained. In Figure 3, it is interesting to see the different capacitive and inductive performance depending on the region of the device at a fixed frequency, although the device is quite symmetric. I suggest including the same figures for the case of dark and check the capacitive/inductive behaviours. Maybe, this new Figure can shed light, where does it come from the different capacitive/inductive performance of each region.`

The profiles in figure 3 are very interesting and contain a lot of information, but these plots have to be carefully analyzed. The influence of a large phase shift might e.g. be neglectable if the corresponding amplitude is small. Additionally, these plots do not tell us anything about the causality. We focused on the contribution plots and the simulations with the ions considered as static background charge as we consider these to be easier applicable to simulations of other devices, especially more involved ones.
Nonetheless, we have added the profiles of the AC potential for the simulation in the dark to the SI. The difference to the plots in figure 3 is very small. This highlights the crucial role of the increased electronic charge carrier density for the illumination-dependent effects.

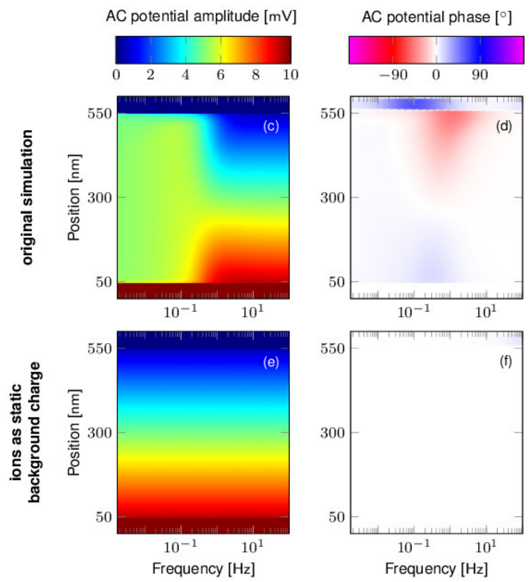

Illuminated (Figure 3)

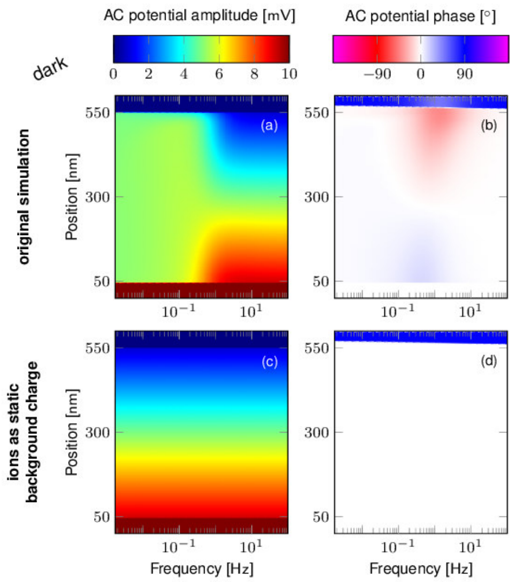

Dark (Figure S3)

I agree with you that the most essential mechanism which originates a high capacitance at low frequency is the electronic-ionic coupling as you are clearly presenting in Figure 2 in the manuscript. However, from my point of view, from your Figure S6, the recombination is substantially promoting and impacting on the magnitude of the capacitance and the frequency onset (https://doi.org/10.1063/5.0216983 in the SI Figure S43 and S46).
In the main text, you have written: "In contrast to previous studies, 12,13 the recombination, despite influencing the EIS results, is not causative for the large capacitance at low frequencies for the studied devices, as the effects persist even without considering any recombination mechanisms (figure S6(e) in the supporting information)." I am still thinking that is a strong statement to say, "effects persist even without considering any recombination mechanisms". From my point of view, the origin of the high capacitance at low frequency is the electronic-ionic coupling and its magnitude is promoted by the recombination.

We see that our wording might be a bit too forthright. We tried to tone it down and hope we find eachother with the following text:

In contrast to previous studies, the recombination is not causative for the large capacitance at low frequencies for the studied devices, as the effects do not vanish even if no recombination mechanisms are considered (figure S6(e) in the supporting information). However, recombination significantly increases the magnitude of the effects.

All changes are marked in the annexed version for review of both the manuscript and the supporting information. The newly added sections in the SI are indicated by a highlighted section title.

Yours sincerely,

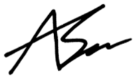

Andreas Schiller
